# Supplementary material for: Genetic Variants in Antineutrophil Cytoplasmic Antibody-Associated Vasculitis: A Bayesian Approach and Systematic Review
Source: J Clin Med. 2019 Feb 21;8(2):266. doi: 10.3390/jcm8020266 (PMC6406345; doi:10.3390/jcm8020266)
Supplement: Supplementary file 1 [file jcm-08-00266-s001.pdf]

Genetic Variants in Antineutrophil Cytoplasmic Antibody-Associated Vasculitis: A Bayesian Approach and Systematic Review

Table S1. Preferred Reporting Items for Systematic Reviews and Meta-Analyses (PRISMA) Checklist.

Section/topic	#	Checklist item	Reported on page #
**TITLE**			
Title	1	Identify the report as a systematic review, meta-analysis, or both.	1
**ABSTRACT**			
Structured summary	2	Provide a structured summary including, as applicable: background; objectives; data sources; study eligibility criteria, participants, and interventions; study appraisal and synthesis methods; results; limitations; conclusions and implications of key findings; systematic review registration number.	3-4
**INTRODUCTION**			
Rationale	3	Describe the rationale for the review in the context of what is already known.	5-6
Objectives	4	Provide an explicit statement of questions being addressed with reference to participants, interventions, comparisons, outcomes, and study design (PICOS).	5-6
**METHODS**			
Protocol and registration	5	Indicate if a review protocol exists, if and where it can be accessed (e.g., Web address), and, if available, provide registration information including registration number.	7-8
Eligibility criteria	6	Specify study characteristics (e.g., PICOS, length of follow-up) and report characteristics (e.g., years considered, language, publication status) used as criteria for eligibility, giving rationale.	7-8
Information sources	7	Describe all information sources (e.g., databases with dates of coverage, contact with study authors to identify additional studies) in the search and date last searched.	7-8
Search	8	Present full electronic search strategy for at least one database, including any limits used, such that it could be repeated.	7-8
Study selection	9	State the process for selecting studies (i.e., screening, eligibility, included in systematic review, and, if applicable, included in the meta-analysis).	7-8 (Figure1)
Data collection process	10	Describe method of data extraction from reports (e.g., piloted forms, independently, in duplicate) and any processes for obtaining and confirming data from investigators.	7-8
Data items	11	List and define all variables for which data were sought (e.g., PICOS, funding sources) and any assumptions and simplifications made.	Tables and Supplementary

			Tables
Risk of bias in individual studies	12	Describe methods used for assessing risk of bias of individual studies (including specification of whether this was done at the study or outcome level), and how this information is to be used in any data synthesis.	N/A
Summary measures	13	State the principal summary measures (e.g., risk ratio, difference in means).	7-8
Synthesis of results	14	Describe the methods of handling data and combining results of studies, if done, including measures of consistency (e.g., I^2) for each meta-analysis.	7-8

Section/topic	#	Checklist item	Reported on page #
Risk of bias across studies	15	Specify any assessment of risk of bias that may affect the cumulative evidence (e.g., publication bias, selective reporting within studies).	Tables and Supplementary Tables
Additional analyses	16	Describe methods of additional analyses (e.g., sensitivity or subgroup analyses, meta-regression), if done, indicating which were pre-specified.	N/A
**RESULTS**			
Study selection	17	Give numbers of studies screened, assessed for eligibility, and included in the review, with reasons for exclusions at each stage, ideally with a flow diagram.	Figure 1
Study characteristics	18	For each study, present characteristics for which data were extracted (e.g., study size, PICOS, follow-up period) and provide the citations.	Tables and Supplementary Tables
Risk of bias within studies	19	Present data on risk of bias of each study and, if available, any outcome level assessment (see item 12).	N/A
Results of individual studies	20	For all outcomes considered (benefits or harms), present, for each study: (a) simple summary data for each intervention group (b) effect estimates and confidence intervals, ideally with a forest plot.	9–11, Tables,
Synthesis of results	21	Present results of each meta-analysis done, including confidence intervals and measures of consistency.	9–11. Tables,
Risk of bias across studies	22	Present results of any assessment of risk of bias across studies (see Item 15).	Tables and Supplementary Tables
Additional analysis	23	Give results of additional analyses, if done (e.g., sensitivity or subgroup analyses, meta-regression [see Item 16]).	N/A
**DISCUSSION**			
Summary of evidence	24	Summarize the main findings including the strength of evidence for each main outcome; consider their relevance to key groups (e.g., healthcare providers, users, and policy makers).	12-16
Limitations	25	Discuss limitations at study and outcome level (e.g., risk of bias), and at review-level (e.g., incomplete retrieval of identified research, reporting bias).	15-16

Conclusions	26	Provide a general interpretation of the results in the context of other evidence, and implications for future research.	12-16
**FUNDING**			
Funding	27	Describe sources of funding for the systematic review and other support (e.g., supply of data); role of funders for the systematic review.	16

Equation S1. Calculation of False Positive Report Probability (FPRP) and Bayesian False Discovery Probability (BFDP)

Estimation of False Positive Report Probability (FPRP), which is defined as "the probability of no true association between a gene variant and disease (null hypothesis)" for a statistically significant association was calculated following methods created by Wacholder et al. FPRP is calculated with the observed p-value, statistical power of the test, and prior probability that a molecular association is real. In this review, we calculated FPRP at two levels of assumed prior probabilities, which were 10^{-3} for a candidate gene variant and 10^{-6} for a random SNP. Thus, readers could judge by themselves about the supporting evidence for a given locus. Statistical power to detect an OR of 1.2 and 1.5 were used for FPRP at both prior probabilities. Though Wacholder et al. suggested estimating statistical power based on the ability to detect an OR of 1.5 (or its reciprocal 1/1.5 = 0.67) for OR less than 1.0, we found this estimate might be too conservative, so we decided to present results for both OR of 1.5 and 1.2 (or its reciprocal 1/1.2 = 0.83), which is the median reported OR in our review. FPRP value can be obtained using the following equation:

$$\mathrm{FPRP} = \frac{\alpha(1-\pi)}{\alpha(1-\pi)+(1-\beta)\pi} \quad (1)$$

where π is the prior probability, α is the lowest level of significance at which a test is noteworthy (α =0.05), and $(1-\beta)$ is the statistical power obtained using the following equation:

$$1-\beta = \varphi\left\{\frac{[\log(OR_A/OR_O)]}{\sigma}\right\} - Z_{\alpha/2} \quad (2)$$

where φ is the cumulative distribution function of the standard normal distribution, and $Z_{\alpha/2}$ is the $\alpha/2$ point of the standard cumulative normal distribution. For the actual computation of FPRP, σ and $Z_{\alpha/2}$ are replaced by the standard error of the log-odds ratio estimates and the two-sided p-value point of the standard normal distribution. Computed FPRP for significant associations are summarized in Tables 1–4. All FPRP computations were performed using the Excel spreadsheet provided by Wacholder et al., and associations with FPRP <0.2 were considered noteworthy as recommended by the authors.

Moreover, we also computed the Bayesian false discovery probability (BFDP) values for the above significant associations using methods created by Wakefield. Unlike FPRP, BFDP is calculated using the following equation:

$$\mathrm{BFDP} = \frac{ABF \times PO}{ABF \times PO + 1} \quad (3)$$

where PO is the prior odds of the null hypothesis and is equal to $\pi_0 / (1 - \pi_0)$ wherein π_0 is the prior probability of the null, and ABF is the approximate Bayes factor computed using odds ratio and standard error. One of the differences between BFDP and FPRP is the independence of BFDP from a statistical power. Its approximation is based on a logistic regression model instead of standard normal distribution.

Even though Wakefield advocates that BFDP is superior to FPRP with its straightforward and sound methodological derivation, Wakefield admits that the overall behavior of BFDP and FPRP is similar. FPRP produces posterior null estimates smaller than those produced by BFDP because the FPRP is essentially the lower bound on the posterior probability corresponding to the observed estimates [1]. Noteworthy genetic variants identified by our analysis are given in Tables 1–4, and more noteworthy genetic variants were derived from BFDP than from FPRP. Similar to the

FPRP computation, BFDP estimation was computed for both prior probabilities of 10^{-3} and 10^{-6} and is shown in Tables 1–4. All BFDP computations were also performed using the Excel spreadsheet provided by Wakefield (http://faculty.washington/edu/jonno/cv.html).

Table S2. List of publications included in this re-analysis [1-7].

No.	Title	Journal publication	Author	Year	Type
1	**Association of granulomatosis with polyangiitis (Wegener's) with HLA-DPB1*04 and SEMA6A gene variants: evidence from genome-wide analysis**	Arthritis Rheum.	Xie G, et al.	2013	GWAS
2	**Identification of Functional and Expression Polymorphisms Associated With Risk for Antineutrophil Cytoplasmic Autoantibody-Associated Vasculitis**	Arthritis Rheumatol.	Merkel PA, et al.	2017	GWAS
3	**CTLA-4 and TNF-α promoter-308 A/G polymorphisms and ANCA-associated vasculitis susceptibility: a meta-analysis**	Mol Biol Rep.	Lee YH, et al.	2012	Meta-analysis
4	**Genetic variants in ANCA-associated vasculitis: a meta-analysis**	Ann Rheum Dis.	Rahmattulla C, et al.	2016	Meta-analysis
5	**Meta-Analysis of Associations Between Interleukin-10 Polymorphisms and Susceptibility to Vasculitis**	Immunol Invest.	Jung JH, et al.	2015	Meta-analysis
6	**Meta-analysis of genetic polymorphisms in granulomatosis with polyangiitis (Wegener's) reveals shared susceptibility loci with rheumatoid arthritis**	Arthritis Rheum.	Chung SA, et al.	2012	Meta-analysis
7	**The protein tyrosine phosphatase nonreceptor 22 C1858T polymorphism and vasculitis: a meta-analysis**	Mol Biol Rep.	Lee YH, et al.	2012	Meta-analysis

Table S3. Meta-analysis results of observational studies, gene variants with statistical significance (p-value < 0.05) for each clinical diagnosis (AAV, GPA, MPA). Summary of genetic variants with FPRP, BFDP values, and noteworthy genetic variants are given in bold.

Gene/Variant	Minor allele/ Comparison	OR (95% CI)	p-value for meta-analysis	Publications (n)	*Diagnosis (clinical subtypes)*	*No. of Cases/Controls*	I^2 (%)	I^2 (P)	Egger's P-value	Power OR 1.2	Power OR 1.5	FPRP Values at Prior probability OR 1.2 0.001	FPRP OR 1.2 0.000001	FPRP OR 1.5 0.001	FPRP OR 1.5 0.000001	BFDP 0.001	BFDP 0.000001	Author, Year
**CD226 rs763361**	**T**	1.14 (1.07 – 1.21)	<0.001	3	*AAV*	2422/17898	0	0.444	0.792	0.954	1.000	**0.017**	0.945	**0.016**	0.942	**0.437**	0.999	Rahmattulla, et al. 2016

																		[4]
CLTA-4 rs231775	G	1.16 (1.06 – 1.28)	0.002	3	*AAV*	1002/6179	60	0.080	0.080	0.750	1.000	0.806	1.000	0.757	1.000	0.986	1.000	Rahmattulla, et al. 2016 [4]
**CTLA-4 rs3087243 (CT60)**	**A**	0.81 (0.75 – 0.87)	<0.001	3	*AAV*	2015/7855	25	0.262	0.122	0.218	1.000	**0.000**	**0.033**	**0.000**	**0.007**	**0.001**	**0.347**	Rahmattulla, et al. 2016 [4]
**CTLA-4**	**(AT)$_{86}$**	0.54 (0.43 – 0.67)	<0.001	4	*AAV*	303/543	89	<0.001	0.946	0.000	0.028	0.348	0.998	**0.001**	0.437	**0.010**	0.913	Rahmattulla, et al. 2016 [4]
CTLA-4 rs231775	GG vs. GA+AA	1.785 (1.275 – 2.499)	0.001	2	*AAV*	*324/817*	0	0.419		0.430	0.682	0.999	1.000	0.999	1.000	0.999	1.000	Lee, et al. 2012 [3]
CTLA-4 rs231775	GG+GA vs. AA	1.369 (1.045 – 1.793)	0.022	2	*AAV*	*324/817*	56	0.128		0.169	0.747	0.993	1.000	0.968	1.000	0.997	1.000	Lee, et al. 2012 [3]
CTLA-4 rs231775	GG vs. AA	1.984 (1.366 – 2.881)	3.17E-04	2	*AAV*	*324/817*	0	0.937		0.004	0.071	0.987	1.000	0.818	0.998	0.978	1.000	Lee, et al. 2012 [3]
CTLA-4 rs231775	G vs. A	1.382 (1.147 – 1.664)	0.001	2	*AAV*	*324/817*	0	0.484		0.068	0.806	0.904	0.999	0.441	0.988	0.951	1.000	Lee, et al. 2012 [3]
CTLA-4 rs3087243 (CT60)	AA vs. AG+GG	0.720 (0.591 – 0.877)	0.001	2	*AAV*	*797/9669*	10.9	0.289		0.073	0.778	0.937	0.999	0.585	0.993	0.969	1.000	Lee, et al. 2012 [3]
CTLA-4 rs3087243 (CT60)	AA+AG vs. GG	0.725 (0.538 – 0.977)	0.035	2	*AAV*	*797/9669*	63.5	0.098		0.180	0.709	0.995	1.000	0.980	1.000	0.997	1.000	Lee, et al. 2012 [3]
**CTLA-4 rs3087243 (CT60)**	**AA vs. GG**	0.693 (0.512 – 0.796)	6.39E-05	2	*AAV*	*797/9669*	57.7	0.124		0.005	0.708	**0.045**	0.824	**0.000**	**0.029**	**0.018**	0.948	Lee, et al. 2012 [3]
CTLA-4 rs3087243 (CT60)	A vs. G	0.769 (0.619 – 0.955)	0.017	2	*AAV*	*797/9669*	67.1	0.081		0.234	0.902	0.987	1.000	0.951	0.999	0.996	1.000	Lee, et al. 2012 [3]
FCGRZA rs 1801274	C	0.90 (0.82 – 0.99)	0.028	6	*AAV*	1239/6209	0	0.834	0.788	0.943	1.000	0.970	1.000	0.968	1.000	0.998	1.000	Rahmattulla, et al. 2016 [4]
HLA-B5	-	0.59 (0.38 – 0.92)	0.019	2	*AAV*	335/6573	0	0.432	NA	0.064	0.295	0.997	1.000	0.985	1.000	0.997	1.000	Rahmattulla, et al. 2016 [4]
HLA-B8	-	1.48 (1.04 – 2.11)	0.028	6	*AAV*	475/7855	47	0.096	0.063	0.123	0.530	0.996	1.000	0.983	1.000	0.998	1.000	Rahmattulla, et al. 2016 [4]
**HLA-DPA1 rs9277341**	**C**	0.35 (0.30 – 0.40)	<0.001	2	*AAV*	1032/2200	54	0.116	0.215	NA	NA	**NA**	**NA**	**NA**	**NA**	**0.000**	**0.000**	Rahmattulla, et al. 2016 [4]
HLA-DPB1*0301	-	0.38 (0.21 – 0.69)	0.002	5	*AAV*	1154/1337	78	<0.001	0.938	0.005	0.032	0.997	1.000	0.979	1.000	0.996	1.000	Rahmattulla, et al. 2016 [4]
HLA-DPB1*0401	-	1.99 (1.44 – 2.74)	<0.001	5	*AAV*	1154/1337	84	<0.001	0.738	0.001	0.042	0.962	1.000	0.373	0.998	0.870	1.000	Rahmattulla, et al. 2016 [4]
**HLA-DPB2 rs3130215**	**A**	1.40 (1.29 – 1.52)	<0.001	3	*AAV*	1417/7249	99	<0.001	0.446	0.000	0.950	**0.000**	**0.000**	**0.000**	**0.000**	**0.000**	**0.000**	Rahmattulla, et al. 2016 [4]
HLA-DPQ1*0303	-	1.82 (1.09 – 3.03)	0.021	3	*AAV*	176/218	17	0.301	0.916	0.055	0.229	0.997	1.000	0.989	1.000	0.998	1.000	Rahmattulla, et al. 2016 [4]
HLA-DR6	-	0.50 (0.27 – 0.95)	0.033	5	*AAV*	487/6222	55	0.062	0.997	0.059	0.190	0.998	1.000	0.994	1.000	0.998	1.000	Rahmattulla, et al. 2016 [4]
HLA-DRB1*1101	-	1.89 (1.15 – 3.08)	0.011	2	*AAV*	268/465	0	0.487	NA	0.034	0.177	0.997	1.000	0.984	1.000	0.997	1.000	Rahmattulla, et al. 2016 [4]
HLA-DRB1*1201	-	0.37 (0.15 – 0.91)	0.031	2	*AAV*	216/465	0	0.491	NA	0.039	0.100	0.999	1.000	0.997	1.000	0.999	1.000	Rahmattulla, et al. 2016 [4]
HLA-DRB1*13	-	0.47 (0.32 – 0.70)	<0.001	4	*AAV*	233/833	0	0.504	0.884	0.002	0.043	0.988	1.000	0.826	1.000	0.977	1.000	Rahmattulla, et al. 2016 [4]
HLA-DRB1*14	-	1.91 (1.07 – 3.42)	0.029	4	*AAV*	322/862	0	0.728	0.700	0.059	0.208	0.998	1.000	0.993	1.000	0.998	1.000	Rahmattulla, et al. 2016 [4]
HLA-DRB1*15	-	1.86 (1.39 – 2.50)	<0.001	3	*AAV*	236/633	69	0.021	0.347	0.002	0.077	0.955	1.000	0.336	0.998	0.871	1.000	Rahmattulla, et al. 2016 [4]
HLA-DRB1*1501	-	1.68 (1.20 – 2.34)	0.002	2	*AAV*	216/465	0	0.925	NA	0.023	0.251	0.989	1.000	0.895	1.000	0.989	1.000	Rahmattulla, et al. 2016 [4]
HLA-DRB3	-	0.62 (0.49 – 0.79)	<0.001	4	*AAV*	260/1845	68	0.024	0.689	0.008	0.279	0.929	1.000	0.283	0.997	0.888	1.000	Rahmattulla, et al. 2016 [4]
**HLA-DRB4**	**-**	1.69 (1.36 – 2.10)	<0.001	4	*AAV*	260/1845	61	0.055	0.533	0.001	0.141	0.686	1.000	**0.015**	0.940	**0.259**	0.997	Rahmattulla, et al. 2016 [4]
**HSD17B8 rs421446**	**C**	0.40 (0.34 – 0.48)	<0.001	2	*AAV*	738/1872	0	0.620	NA	NA	NA	**NA**	**NA**	**NA**	**NA**	**0.000**	**0.000**	Rahmattulla, et al. 2016 [4]
**IRF5 rs10954213**	**G**	0.77 (0.70 – 0.83)	<0.001	3	*AAV*	1535/6977	99	<0.001	0.948	0.019	1.000	**0.000**	**0.000**	**0.000**	**0.000**	**0.000**	**0.001**	Rahmattulla, et al. 2016 [4]
**PTPN22 rs2476601**	**T vs. C**	1.415 (1.228 – 1.630)	1.59E-06	3	*AAV*	*1184/10459*	0	0.393	0.481	0.011	0.791	**0.119**	0.931	**0.002**	**0.160**	**0.091**	0.990	Lee, et al. 2012 [7]
**PTPN22 rs2476601**	**A**	1.39 (1.24 – 1.56)	<0.001	4	*AAV*	2099/8678	0	0.693	0.500	0.006	0.902	**0.004**	0.780	**0.000**	**0.024**	**0.002**	**0.654**	Rahmattulla, et al. 2016 [4]
**RING1/RXRB rs213213**	**A**	1.71 (1.57 – 1.86)	<0.001	3	*AAV*	1414/7238	73	0.026	0.187	NA	NA	**NA**	**NA**	**NA**	**NA**	**0.000**	**0.000**	Rahmattulla, et al. 2016 [4]
**RXRB rs6531**	**C**	1.63 (1.50 – 1.77)	<0.001	3	*AAV*	1557/6955	96	<0.001	0.292	NA	NA	**NA**	**NA**	**NA**	**NA**	**0.000**	**0.000**	Rahmattulla, et al. 2016 [4]
**RXRB rs9277935**	**T**	0.44 (0.37 – 0.50)	<0.001	3	*AAV*	1417/7233	73	0.025	0.393	NA	NA	**NA**	**NA**	**NA**	**NA**	**0.000**	**0.000**	Rahmattulla, et al. 2016 [4]
SERPINA 1	S allele	1.30 (1.03 – 1.63)	0.025	5	*AAV*	1474/5762	0	0.464	0.547	0.244	0.892	0.989	1.000	0.963	1.000	0.997	1.000	Rahmattulla, et al. 2016

																		[4]
**SERPINA 1**	**Z allele**	2.94 (2.22 – 3.88)	<0.001	8	*AAV*	3662/8581	41	0.092	0.078	0.000	0.000	**0.173**	0.995	**0.000**	**0.025**	**0.000**	**0.005**	Rahmattulla, et al. 2016 [4]
STAT4 rs 7574865	T	1.11 (1.01 – 1.22)	0.029	3	*AAV*	1520/6956	3	0.357	0.590	0.947	1.000	0.970	1.000	0.968	1.000	0.998	1.000	Rahmattulla, et al. 2016 [4]
TLR9 rs 352139	T	1.11 (1.00 – 1.23)	0.041	1	*AAV*	1289/1898	0	0.756	NA	0.932	1.000	0.980	1.000	0.979	1.000	0.998	1.000	Rahmattulla, et al. 2016 [4]
TLR9 rs 352140	T	1.13 (1.02 – 1.25)	0.018	1	*AAV*	1289/1898	0	0.432	NA	0.878	1.000	0.952	1.000	0.946	1.000	0.997	1.000	Rahmattulla, et al. 2016 [4]
**TLR9 rs352162**	**T**	1.58 (1.43 – 1.75)	<0.001	1	*AAV*	1289/1898	96	<0.001	NA	NA	NA	**NA**	**NA**	**NA**	**NA**	**0.000**	**0.000**	Rahmattulla, et al. 2016 [4]
**PTPN22 rs2476601**	**T vs. C**	2.042 (1.534 – 2.719)	1.02E-06	2	*ANCA(+) GPA*	-	0	0.989	NA	0.000	0.017	0.882	0.999	**0.056**	0.855	**0.375**	0.998	Lee, et al. 2012 [7]
BACH2 rs11755527	G vs. C	1.14 (1.01 – 1.28)	0.03	2	*GPA*	*880/1969*				0.807	1.000	0.971	1.000	0.964	1.000	0.997	1.000	Chung, et al. 2012 [6]
**CD226 rs763361**	**T**	1.19 (1.11 – 1.28)	<0.001	3	*GPA*	*2021/17898*	72.2	0.006		0.589	1.000	**0.005**	0.832	**0.003**	0.745	**0.124**	0.993	Rahmattulla, et al. 2016 [4]
CD40 rs4810485	T vs. G/A	0.81 (0.70 – 0.92)	0.002	2	*GPA*	*880/1969*				0.331	0.999	0.781	0.997	0.542	0.992	0.965	1.000	Chung, et al. 2012 [6]
**CLTA-4 rs3087243**	**A**	0.80 (0.73 – 0.87)	<0.001	3	*GPA*	*1561/7855*	38.7	0.180		0.170	1.000	**0.001**	0.521	**0.000**	**0.156**	**0.011**	0.915	Rahmattulla, et al. 2016 [4]
**CTLA-4**	**(*AT*)$_{86}$**	0.44 (0.34 – 0.57)	<0.001	3	*GPA*	*210/432*	86.5	0.001		0.000	0.001	0.434	0.999	**0.001**	0.381	**0.002**	**0.670**	Rahmattulla, et al. 2016 [4]
CTLA-4	(AT)$_{86}$ vs. xx	0.402 (0.184 – 0.875)	0.022	3	*GPA*	*251/420*	81.6	<0.001		0.033	0.101	0.998	1.000	0.995	1.000	0.998	1.000	Lee, et al. 2012 [3]
CTLA-4	(AT)$_{86}$/(AT)$_{86}$ vs. (AT)$_{86}$/xx+xx/xx	0.376 (0.156 – 0.908)	0.030	3	*GPA*	*251/420*	80.0	0.007		0.038	0.101	0.999	1.000	0.997	1.000	0.999	1.000	Lee, et al. 2012 [3]
CTLA-4	(AT)$_{86}$/(AT)$_{86}$+(AT)$_{86}$/xx vs. xx/xx	0.428 (0.221 – 0.827)	0.012	3	*GPA*	*251/420*	70.9	0.032		0.024	0.094	0.998	1.000	0.992	1.000	0.998	1.000	Lee, et al. 2012 [3]
CTLA-4 rs231735	G vs. T	0.82 (0.73 – 0.92)	0.001	2	*GPA*	*880/1969*				0.392	1.000	0.649	0.995	0.420	0.986	0.949	1.000	Chung, et al. 2012 [6]
**CTLA-4 rs3087243 (CT60)**	**A vs. G**	0.79 (0.70 – 0.89)	9.83E-05	2	*GPA*	*880/1969*				0.190	0.997	0.358	0.982	**0.096**	0.914	**0.779**	1.000	Chung, et al. 2012 [6]
HLA-A11	-	0.61 (0.41 – 0.90)	0.014	5	*GPA*	*380/7702*	51.4	0.084		0.058	0.327	0.995	1.000	0.975	1.000	0.997	1.000	Rahmattulla, et al. 2016 [4]
HLA-B35	-	0.62 (0.44 – 0.89)	0.010	4	*GPA*	*349/7095*	0.0	0.894		0.054	0.347	0.994	1.000	0.965	1.000	0.996	1.000	Rahmattulla, et al. 2016 [4]
HLA-B62	-	2.27 (1.05 – 4.90)	0.036	2	*GPA*	*57/1172*	0.0	0.927		0.052	0.146	0.999	1.000	0.996	1.000	0.999	1.000	Rahmattulla, et al. 2016 [4]
**HLA-DPA1 rs9277341**	**C**	0.35 (0.30 – 0.41)	<0.001	2	*GPA*	*1032/2200*	54.8	0.109		NA	NA	**NA**	**NA**	**NA**	**NA**	**0.000**	**0.000**	Rahmattulla, et al. 2016 [4]
HLA-DPB1*0101	-	0.17 (0.05 – 0.63)	0.008	1	*GPA*	*148/89*	-	-		0.009	0.020	0.999	1.000	0.997	1.000	0.999	1.000	Rahmattulla, et al. 2016 [4]
HLA-DPB1*0201	-	0.67 (0.45 – 0.99)	0.042	2	*GPA*	*283/458*	40.0	0.197		0.137	0.510	0.997	1.000	0.989	1.000	0.998	1.000	Rahmattulla, et al. 2016 [4]
**HLA-DPB1*0301**	**-**	0.23 (0.16 – 0.32)	<0.001	3	*GPA*	*774/918*	61.7	0.050		NA	NA	**NA**	**NA**	**NA**	**NA**	**0.000**	**0.000**	Rahmattulla, et al. 2016 [4]
**HLA-DPB1*0401**	**-**	2.89 (2.50 – 3.35)	<0.001	3	*GPA*	*774/918*	67.5	0.026		NA	NA	**NA**	**NA**	**NA**	**NA**	**0.000**	**0.000**	Rahmattulla, et al. 2016 [4]
HLA-DR2	-	1.36 (1.08 – 1.72)	0.010	4	*GPA*	*301/6132*	71.1	0.016		0.148	0.793	0.986	1.000	0.928	1.000	0.994	1.000	Rahmattulla, et al. 2016 [4]
HLA-DR4	-	1.32 (1.09 – 1.60)	0.004	7	*GPA*	*425/6424*	0.0	0.461		0.166	0.904	0.966	1.000	0.838	1.000	0.989	1.000	Rahmattulla, et al. 2016 [4]
**HLA-DR6**	**-**	0.45 (0.33 – 0.62)	<0.001	4	*GPA*	*301/6132*	59.8	0.058		0.000	0.008	0.927	1.000	**0.114**	0.992	**0.511**	0.999	Rahmattulla, et al. 2016 [4]
HLA-DRB1*12	-	4.06 (1.27 – 12.99)	0.018	2	*GPA*	*83/142*	0.0	0.553		0.020	0.047	0.999	1.000	0.997	1.000	0.999	1.000	Rahmattulla, et al. 2016 [4]
HLA-DRB1*1202	-	2.92 (1.37 – 6.23)	0.005	1	*GPA*	*45/200*	-	-		0.011	0.042	0.998	1.000	0.992	1.000	0.998	1.000	Rahmattulla, et al. 2016 [4]
IDIN rs9585056	C vs. T	0.85 (0.74 – 0.98)	0.03	2	*GPA*	*880/1969*				0.607	1.000	0.976	1.000	0.962	1.000	0.997	1.000	Chung, et al. 2012 [6]
IL-10 rs1800896	C vs. T	0.729 (0.547 - 0.971)	0.031	2	*GPA*	*164/225*	0	-	0.381	0.180	0.729	0.994	1.000	0.977	1.000	0.997	1.000	Jung, et al. 2015 [5]
IL27 rs4788084	T vs. C	1.18 (1.05 – 1.33)	0.006	2	*GPA*	*880/1969*				0.608	1.000	0.917	0.999	0.870	0.999	0.992	1.000	Chung, et al. 2012 [6]
IL27, others rs151181	C vs. T	1.18 (1.05 – 1.34)	0.007	2	*GPA*	*880/1969*				0.602	1.000	0.947	0.999	0.915	0.999	0.994	1.000	Chung, et al. 2012 [6]
IL2RA rs706778	T vs. C	1.13 (1.00 – 1.27)	0.05	2	*GPA*	*880/1969*				0.843	1.000	0.979	1.000	0.976	1.000	0.998	1.000	Chung, et al. 2012 [6]
**IRF5 rs10954213**	**G**	0.66 (0.59 – 0.74)	<0.001	2	*GPA*	*1021/6267*	99.1	0.000		0.000	0.432	**0.000**	**0.033**	**0.000**	**0.000**	**0.000**	**0.000**	Rahmattulla, et al. 2016 [4]
JAK2 rs10758669	C vs. T/A	1.14 (1.01 – 1.29)	0.03	2	*GPA*	*880/1969*				0.792	1.000	0.979	1.000	0.974	1.000	0.998	1.000	Chung, et al. 2012 [6]
LEPR rs8179183	C	0.72 (0.60 – 0.86)	<0.001	1	*GPA*	*682/1326*	0.0	0.968		0.053	0.802	0.844	1.000	0.266	0.997	0.910	1.000	Rahmattulla, et al. 2016 [4]

NKX2-3 rs11190140	T vs. C	0.86 (0.77 – 0.97)	0.01	2	*GPA*	*880/1969*				0.696	1.000	0.953	1.000	0.933	0.999	0.995	1.000	Chung, et al. 2012 [6]
NKX2-3 rs6584283	T vs. C	0.86 (0.76 – 0.96)	0.009	2	*GPA*	*880/1969*				0.713	1.000	0.910	0.999	0.878	0.999	0.992	1.000	Chung, et al. 2012 [6]
PARK7, DJ-1 rs3766606	T vs. G	0.80 (0.70 – 0.92)	0.005	2	*GPA*	*880/1969*				0.283	0.995	0.861	0.998	0.638	0.994	0.975	1.000	Chung, et al. 2012 [6]
PARK7, TNFRSF9 rs12727642	A vs. C	0.81 (0.69 – 0.95)	0.01	2	*GPA*	*880/1969*				0.363	0.992	0.963	1.000	0.906	0.999	0.993	1.000	Chung, et al. 2012 [6]
PHRF1, KIAA1542 rs4963128	T vs. C	0.87 (0.77 – 0.99)	0.03	2	*GPA*	*880/1969*				0.743	1.000	0.979	1.000	0.972	1.000	0.998	1.000	Chung, et al. 2012 [6]
**PTPN22 rs2476601**	**A**	1.43 (1.26 – 1.62)	<0.001	4	*GPA*	*1616/8678*	0.0	0.411		0.003	0.774	**0.006**	0.867	**0.000**	**0.024**	**0.002**	**0.649**	Rahmattulla, et al. 2016 [4]
PTPN22 rs2476601	T vs. C	1.829 (1.377 – 2.431)	3.09E-05	2	*GPA*	-	0	0.804	NA	0.002	0.086	0.945	0.999	0.271	0.974	0.841	1.000	Lee, et al. 2012 [7]
PTPN22 rs2476601	T vs. C	1.35 (1.12 – 1.62)	0.002	2	*GPA*	*880/1969*				0.103	0.871	0.924	0.999	0.590	0.993	0.970	1.000	Chung, et al. 2012 [6]
RASGRP1 rs17574546	C vs. A	1.18 (1.02 – 1.36)	0.03	2	*GPA*	*880/1969*				0.592	1.000	0.974	1.000	0.957	1.000	0.997	1.000	Chung, et al. 2012 [6]
**RING1/RXRB rs213213**	**A**	1.91 (1.73 – 2.10)	<0.001	3	*GPA*	*1132/7238*	0.0	0.551		NA	NA	**NA**	**NA**	**NA**	**NA**	**0.000**	**0.000**	Rahmattulla, et al. 2016 [4]
**RXRB rs6531**	**C**	1.70 (1.55 – 1.86)	<0.001	3	*GPA*	*1211/6955*	96.5	0.000		NA	NA	**NA**	**NA**	**NA**	**NA**	**0.000**	**0.000**	Rahmattulla, et al. 2016 [4]
**RXRB rs9277935**	**T**	0.37 (0.31 – 0.43)	<0.001	3	*GPA*	*1135/7233*	0.0	0.798		NA	NA	**NA**	**NA**	**NA**	**NA**	**0.000**	**0.000**	Rahmattulla, et al. 2016 [4]
SERPINA 1	S allele	1.72 (1.12 – 2.66)	0.014	2	*GPA*	*484/1079*	0.0	0.588		0.053	0.269	0.996	1.000	0.982	1.000	0.997	1.000	Rahmattulla, et al. 2016 [4]
**SERPINA 1**	**Z allele**	2.40 (1.73 - 3.33)	<0.001	4	*GPA*	*972/2636*	0.0	0.763		0.000	0.002	0.906	1.000	**0.062**	0.985	**0.282**	0.997	Rahmattulla, et al. 2016 [4]
TLR9 rs352139	T	1.18 (1.06 – 1.32)	0.003	1	*GPA*	*919/1898*	0.0	0.366		0.616	1.000	0.861	1.000	0.792	1.000	0.987	1.000	Rahmattulla, et al. 2016 [4]
TLR9 rs352140	T	1.20 (1.07 – 1.35)	0.001	1	*GPA*	*919/1898*	39.7	0.198		0.500	1.000	0.828	1.000	0.707	1.000	0.981	1.000	Rahmattulla, et al. 2016 [4]
TLR9 rs352162	T	1.20 (1.07 – 1.34)	0.001	1	*GPA*	*919/1898*	56.9	0.128		0.500	1.000	0.706	1.000	0.546	0.999	0.966	1.000	Rahmattulla, et al. 2016 [4]
TLR9 rs5743836	G	0.83 (0.70 – 0.99)	0.037	1	*GPA*	*919/1898*	70.5	0.066		0.482	0.993	0.988	1.000	0.975	1.000	0.998	1.000	Rahmattulla, et al. 2016 [4]
FCGR3B	NA1	1.52 (1.03 – 2.25)	0.035	2	*MPA*	*75/200*	0.0	0.774		0.119	0.474	0.997	1.000	0.987	1.000	0.998	1.000	Rahmattulla, et al. 2016 [4]
HLA-A11	-	2.97 (1.19 – 7.41)	0.020	1	*MPA*	*23/405*	-	-		0.026	0.072	0.999	1.000	0.996	1.000	0.999	1.000	Rahmattulla, et al. 2016 [4]
HLA-A26	-	2.49 (1.00 – 6.18)	0.049	1	*MPA*	*23/405*	-	-		0.058	0.137	0.999	1.000	0.997	1.000	0.999	1.000	Rahmattulla, et al. 2016 [4]
HLA-DPB2 rs3130215	A	1.33(1.06 – 1.66)	0.013	1	*MPA*	*156/5366*	-	-		0.182	0.856	0.985	1.000	0.932	1.000	0.994	1.000	Rahmattulla, et al. 2016 [4]
HLA-DQB1*0303	-	2.11 (1.14 – 3.90)	0.018	1	*MPA*	*50/77*	-	-		0.036	0.138	0.998	1.000	0.992	1.000	0.998	1.000	Rahmattulla, et al. 2016 [4]
HLA-DR6	-	0.29 (0.09 – 0.92)	0.035	1	*MPA*	*30/5442*	-	-		0.037	0.079	0.999	1.000	0.998	1.000	0.999	1.000	Rahmattulla, et al. 2016 [4]
HLA-DRB1*1101	-	2.57 (1.56 – 4.23)	<0.001	2	*MPA*	*223/465*	0.0	0.885		0.001	0.017	0.993	1.000	0.923	1.000	0.988	1.000	Rahmattulla, et al. 2016 [4]
HLA-DRB1*1501	-	1.65 (1.14 – 2.38)	0.007	2	*MPA*	*157/465*	0.0	0.805		0.044	0.305	0.994	1.000	0.960	1.000	0.995	1.000	Rahmattulla, et al. 2016 [4]
IRF5 rs10954213	G	1.22 (1.03 – 1.44)	0.018	2	*MPA*	*333/6075*	0.0	0.406		0.423	0.993	0.978	1.000	0.950	1.000	0.996	1.000	Rahmattulla, et al. 2016 [4]
PTPN22 rs2476601	A	1.42 (1.06 – 1.89)	0.018	2	*MPA*	*258/6310*	0.0	0.641		0.124	0.646	0.992	1.000	0.962	1.000	0.996	1.000	Rahmattulla, et al. 2016 [4]
RXRB rs6531	C	1.38 (1.15 – 1.66)	0.001	1	*MPA*	*262/5251*	-	-		0.069	0.812	0.901	1.000	0.438	0.999	0.950	1.000	Rahmattulla, et al. 2016 [4]
TLR9 rs352139	T	0.68 (0.52 – 0.87)	0.003	1	*MPA*	*153/1898*	86.6	0.006		0.053	0.563	0.976	1.000	0.793	1.000	0.984	1.000	Rahmattulla, et al. 2016 [4]
TLR9 rs352140	T	0.71 (0.55 – 0.91)	0.006	1	*MPA*	*153/1898*	71.4	0.062		0.103	0.691	0.985	1.000	0.908	1.000	0.993	1.000	Rahmattulla, et al. 2016 [4]
TLR9 rs352162	T	0.74 (0.58 – 0.95)	0.020	1	*MPA*	*153/1898*	88.0	0.004		0.176	0.794	0.990	1.000	0.958	1.000	0.996	1.000	Rahmattulla, et al. 2016 [4]
TLR9 rs5743836	G	1.78 (1.32 – 2.39)	<0.001	1	*MPA*	*153/1898*	0.0	0.927		0.004	0.127	0.966	1.000	0.496	0.999	0.932	1.000	Rahmattulla, et al. 2016 [4]
HLA-DPB1*0301	-	0.47 (0.29 – 0.75)	0.001	2	*EGPA*	*167/829*	72.1	0.028		0.008	0.071	0.995	1.000	0.956	1.000	0.994	1.000	Rahmattulla, et al. 2016 [4]
HLA-DR4	-	1.70 (1.23 – 2.34)	0.001	4	*EGPA*	*176/6246*	0.0	0.820		0.016	0.221	0.986	1.000	0.837	1.000	0.984	1.000	Rahmattulla, et al. 2016 [4]

HLA-DR6	-	0.19 (0.04 – 0.95)	0.043	2	*EGPA*	*26/5555*	0.0	0.724		0.036	0.063	0.999	1.000	0.999	1.000	0.999	1.000	Rahmattulla, et al. 2016 [4]
HLA-DR7	-	1.71 (1.24 – 2.37)	0.001	3	*EGPA*	*164/804*	58.0	0.093		0.017	0.216	0.987	1.000	0.855	1.000	0.986	1.000	Rahmattulla, et al. 2016 [4]
HLA-DR8	-	3.08 (1.75 – 5.41)	<0.001	2	*EGPA*	*114/5811*	91.2	0.001		0.001	0.006	0.994	1.000	0.936	1.000	0.989	1.000	Rahmattulla, et al. 2016 [4]
HLA-DRB1*13	-	0.40 (0.24 – 0.68)	0.001	2	*EGPA*	*150/691*	29.2	0.235		0.003	0.030	0.995	1.000	0.960	1.000	0.993	1.000	Rahmattulla, et al. 2016 [4]
HLA-DRB3	-	0.58 (0.45 – 0.76)	<0.001	2	*EGPA*	*150/691*	0.0	0.653		0.004	0.156	0.948	1.000	0.333	0.998	0.890	1.000	Rahmattulla, et al. 2016 [4]
**HLA-DRB4**	**-**	2.06 (1.57 – 2.69)	<0.001	2	*EGPA*	*150/691*	0.4	0.316		0.000	0.010	0.754	1.000	**0.011**	0.918	**0.089**	0.990	Rahmattulla, et al. 2016 [4]
IL-10 rs1800896	G	0.68 (0.50 – 0.92)	0.012	1	*EGPA*	*103/507*	-	-		0.094	0.551	0.992	1.000	0.957	1.000	0.996	1.000	Rahmattulla, et al. 2016 [4]
LEPR rs8179183	C	1.41 (1.10 – 1.81)	0.007	1	*EGPA*	*196/1327*	-	-		0.103	0.686	0.986	1.000	0.911	1.000	0.993	1.000	Rahmattulla, et al. 2016 [4]
TLR9 rs352162	T	1.28 (1.05 – 1.57)	0.015	1	*EGPA*	*217/1898*	0.0	0.410		0.268	0.936	0.985	1.000	0.950	1.000	0.996	1.000	Rahmattulla, et al. 2016 [4]

Table S4. Meta-analysis results of observational studies, gene variants with statistical significance (p-value < 0.05) for each serologic diagnosis (MPO-ANCA, PR3-ANCA). Summary of genetic variants with FPRP, BFDP values, and noteworthy genetic variants are given in bold.

Gene/Variant	Minor allele/ Comparison	OR (95% CI)	p-value for meta-analysis	Publications (n)	*Diagnosis (serologic subtypes)*	*No. of Cases/Controls*	I^2 (%)	I^2 (P)	Egger's P-value	Power OR 1.2	Power OR 1.5	FPRP Values at Prior probability				BFDP 0.001	BFDP 0.000001	Author, Year
												OR 1.2		OR 1.5				
												0.001	0.000001	0.001	0.000001			
CTLA-4	$(AT)_{122}$	3.69 (1.40 – 9.71)	0.008	2	*MPO-ANCA*	*92/311*	0.0	0.326		0.011	0.034	0.999	1.000	0.996	1.000	0.998	1.000	Rahmattulla, et al. 2016 [4]
FCGR2A rs1801274	C	0.80 (0.67 – 0.95)	0.011	4	*MPO-ANCA*	*844/5969*	58.7	0.064		0.321	0.981	0.971	1.000	0.918	1.000	0.994	1.000	Rahmattulla, et al. 2016 [4]
HLA-DPB2 rs3130215	A	1.27 (1.02 – 1.58)	0.032	1	*MPO-ANCA*	*167/5366*	-	-		0.305	0.932	0.991	1.000	0.972	1.000	0.997	1.000	Rahmattulla, et al. 2016 [4]
HLA-DQB1*0303	-	1.91 (1.12 – 3.26)	0.017	2	*MPO-ANCA*	*72/127*	0.0	0.535		0.044	0.188	0.998	1.000	0.989	1.000	0.998	1.000	Rahmattulla, et al. 2016 [4]
HLA-DR4	-	2.51 (1.46 – 4.32)	0.001	2	*MPO-ANCA*	*69/202*	57.0	0.127		0.004	0.032	0.996	1.000	0.966	1.000	0.994	1.000	Rahmattulla, et al. 2016 [4]
HLA-DR9	-	1.83 (1.29 – 2.60)	0.001	3	*MPO-ANCA*	*175/1116*	0.0	0.631		0.009	0.134	0.988	1.000	0.848	1.000	0.983	1.000	Rahmattulla, et al. 2016 [4]
HLA-DRB1*1501	-	2.03 (1.26 – 3.29)	0.004	2	*MPO-ANCA*	*89/465*	0.0	0.398		0.016	0.110	0.996	1.000	0.974	1.000	0.996	1.000	Rahmattulla, et al. 2016 [4]
HLA-DRB1*16	-	3.55 (1.02 – 12.38)	0.047	1	*MPO-ANCA*	*51/491*	0.0	0.658		0.044	0.088	0.999	1.000	0.998	1.000	0.999	1.000	Rahmattulla, et al. 2016 [4]
PTPN22 rs2476601	A	1.47 (1.08 – 2.00)	0.014	2	*MPO-ANCA*	*183/6310*	73.6	0.052		0.098	0.551	0.993	1.000	0.963	1.000	0.996	1.000	Rahmattulla, et al. 2016 [4]
RXRB rs6531	C	1.21 (1.00 – 1.46)	0.046	1	*MPO-ANCA*	*264/5251*	-	-		0.465	0.988	0.990	1.000	0.979	1.000	0.998	1.000	Rahmattulla, et al. 2016 [4]
SERPINA 1	Z allele	2.01 (1.04 - 3.87)	0.037	1	*MPO-ANCA*	*166/805*	-	-		0.061	0.191	0.998	1.000	0.995	1.000	0.998	1.000	Rahmattulla, et al. 2016 [4]
SERPINA 1	Z allele	3.13 (1.21 – 8.13)	0.019	2	*MPO-ANCA*	*78/2510*	26.7	0.243		0.025	0.065	0.999	1.000	0.997	1.000	0.999	1.000	Rahmattulla, et al. 2016 [4]
STAT4 rs7574865	T	1.24 (1.05 – 1.46)	0.009	2	*MPO-ANCA*	*908/6076*	64.0	0.095		0.347	0.989	0.966	1.000	0.909	1.000	0.993	1.000	Rahmattulla, et al. 2016 [4]
TLR9 rs352139	T	0.78 (0.63 – 0.96)	0.017	1	*MPO-ANCA*	*NR/NR*	37.4	0.206		0.266	0.931	0.986	1.000	0.953	1.000	0.996	1.000	Rahmattulla, et al. 2016 [4]
TLR9 rs352140	T	0.75 (0.62 – 0.91)	0.004	1	*MPO-ANCA*	*NR/NR*	0.0	0.471		0.143	0.884	0.961	1.000	0.800	1.000	0.986	1.000	Rahmattulla, et al. 2016 [4]
TLR9 rs352162	T	0.79 (0.65 – 0.97)	0.028	1	*MPO-ANCA*	*NR/NR*	0.0	0.402		0.305	0.947	0.988	1.000	0.963	1.000	0.997	1.000	Rahmattulla, et al. 2016 [4]
CD226 rs763361	T	1.22 (1.09 – 1.36)	<0.001	2	*PR3-ANCA*	*764/6592*	86.6	0.006		0.383	1.000	0.465	0.999	0.250	0.997	0.905	1.000	Rahmattulla, et al. 2016 [4]
CLTA-4 rs3087243	A	0.81 (0.71 – 0.93)	0.003	1	*PR3-ANCA*	*478/5257*	-	-		0.344	0.997	0.890	1.000	0.737	1.000	0.983	1.000	Rahmattulla, et al. 2016 [4]

CTLA-4	$(AT)_{104}$	0.42 (0.22 – 0.79)	0.007	1	*PR3-ANCA*	*62/200*	-	-	0.017	0.076	0.998	1.000	0.989	1.000	0.998	1.000	Rahmattulla, et al. 2016 [4]
HLA-B55	-	7.35 (2.33 – 23.14)	0.001	1	*PR3-ANCA*	*16/472*	-	-	0.001	0.003	0.999	1.000	0.995	1.000	0.998	1.000	Rahmattulla, et al. 2016 [4]
**HLA-DPA1 rs9277341**	**C**	0.27 (0.22 – 0.33)	<0.001	1	*PR3-ANCA*	*578/1820*	-	-	NA	NA	**NA**	**NA**	**NA**	**NA**	**0.000**	**0.000**	Rahmattulla, et al. 2016 [4]
HLA-DPB1*0101	-	0.45 (0.20 – 0.99)	0.048	2	*PR3-ANCA*	*183/139*	74.5	0.048	0.063	0.164	0.999	1.000	0.997	1.000	0.999	1.000	Rahmattulla, et al. 2016 [4]
HLA-DPB1*0201	-	0.44 (0.21 – 0.91)	0.027	1	*PR3-ANCA*	*148/89*	-	-	0.042	0.131	0.998	1.000	0.995	1.000	0.998	1.000	Rahmattulla, et al. 2016 [4]
HLA-DPB1*0301	-	0.19 (0.09 – 0.39)	<0.001	2	*PR3-ANCA*	*183/139*	90.8	0.001	0.000	0.000	0.995	1.000	0.951	1.000	0.990	1.000	Rahmattulla, et al. 2016 [4]
**HLA-DPB1*0401**	**-**	3.93 (2.75 – 5.62)	<0.001	2	*PR3-ANCA*	*183/139*	0.0	0.960	0.000	0.000	0.615	0.999	**0.001**	0.495	**0.000**	**0.170**	Rahmattulla, et al. 2016 [4]
**HLA-DPB2 rs3130215**	**A**	0.65 (0.55 – 0.77)	<0.001	1	*PR3-ANCA*	*326/5366*	-	-	0.002	0.385	0.235	0.997	**0.002**	0.618	**0.062**	0.985	Rahmattulla, et al. 2016 [4]
HLA-DR1	-	0.57 (0.38 – 0.86)	0.008	3	*PR3-ANCA*	*157/560*	0.0	0.506	0.035	0.228	0.995	1.000	0.970	1.000	0.996	1.000	Rahmattulla, et al. 2016 [4]
HLA-DR9	-	3.03 (1.40 – 6.56)	0.005	1	*PR3-ANCA*	*16/472*	-	-	0.009	0.037	0.998	1.000	0.992	1.000	0.998	1.000	Rahmattulla, et al. 2016 [4]
**HLA-DRB1*15**	**-**	2.82 (2.00 – 3.96)	<0.001	2	*PR3-ANCA*	*131/582*	84.0	0.002	0.000	0.000	0.842	1.000	**0.016**	0.942	**0.040**	0.977	Rahmattulla, et al. 2016 [4]
PTPN22 rs2476601	A	1.42 (1.14 – 1.77)	0.002	2	*PR3-ANCA*	*419/6310*	13.2	0.283	0.067	0.687	0.964	1.000	0.725	1.000	0.980	1.000	Rahmattulla, et al. 2016 [4]
**RING1/RXRB rs213213**	**A**	2.06 (1.75 – 2.41)	<0.001	1	*PR3-ANCA*	*326/5366*	-	-	NA	NA	**NA**	**NA**	**NA**	**NA**	**0.000**	**0.000**	Rahmattulla, et al. 2016 [4]
**RXRB rs6531**	**C**	2.19 (1.92 – 2.51)	<0.001	1	*PR3-ANCA*	*478/5251*	-	-	NA	NA	**NA**	**NA**	**NA**	**NA**	**0.000**	**0.000**	Rahmattulla, et al. 2016 [4]
**RXRB rs9277935**	**T**	0.24 (0.17 – 0.33)	<0.001	1	*PR3-ANCA*	*326/5350*	-	-	NA	NA	**NA**	**NA**	**NA**	**NA**	**0.000**	**0.000**	Rahmattulla, et al. 2016 [4]
SERPINA 1	S allele	1.92 (1.12 – 3.29)	0.017	3	*PR3-ANCA*	*145/3836*	86.4	0.001	0.044	0.184	0.998	1.000	0.990	1.000	0.998	1.000	Rahmattulla, et al. 2016 [4]
SERPINA 1	Z allele	2.58 (1.57 - 4.25)	<0.001	1	*PR3-ANCA*	*322/805*	-	-	0.001	0.017	0.993	1.000	0.922	1.000	0.988	1.000	Rahmattulla, et al. 2016 [4]
**SERPINA 1**	**Z allele**	3.53 (2.28 – 5.49)	<0.001	5	*PR3-ANCA*	*280/4788*	21.3	0.279	0.000	0.000	0.963	1.000	0.229	0.997	**0.512**	0.999	Rahmattulla, et al. 2016 [4]
TLR9 rs352139	T	1.23 (1.09 – 1.40)	0.001	1	*PR3-ANCA*	*NR/NR*	0.0	0.910	0.354	0.999	0.829	1.000	0.633	0.999	0.974	1.000	Rahmattulla, et al. 2016 [4]
**TLR9 rs352140**	**T**	1.28 (1.12 – 1.45)	0.018	1	*PR3-ANCA*	*NR/NR*	0.0	0.782	0.155	0.994	0.402	0.999	**0.095**	0.991	**0.778**	1.000	Rahmattulla, et al. 2016 [4]
**TLR9 rs352162**	**T**	1.30 (1.14 – 1.47)	<0.001	1	*PR3-ANCA*	*NR/NR*	0.0	0.503	0.101	0.989	0.221	0.996	**0.028**	0.967	**0.532**	0.999	Rahmattulla, et al. 2016 [4]
TLR9 rs5743836	G	0.83 (0.70 – 1.00)	0.045	1	*PR3-ANCA*	*NR/NR*	81.9	0.019	0.483	0.989	0.990	1.000	0.981	1.000	0.998	1.000	Rahmattulla, et al. 2016 [4]

Table S5. Results of meta analyses (combined analysis) with genome-wide association studies and replication cohort. Noteworthy genetic variants with satisfied FPRP and BFDP values are given in bold.

Gene/Variant	Comparison	OR (95% CI)	p-value	*Diagnosis (clinical/serologic subtypes)*	*Ethnicity*	*No. of Cases/Controls*	Power OR 1.2	Power OR 1.5	FPRP Values at Prior probability				BFDP 0.001	BFDP 0.000001	Author, Year
									OR 1.2		OR 1.5				
									0.001	0.000001	0.001	0.000001			
SNPs statistically significant (P<5.00E-08)															
**HLA-DPA1 rs9277341**	**T vs. C**	2.44 (2.21 – 2.69)	6.09E-71	*AAV*	*Caucasian*	*1986/4723*	NA	NA	**NA**	**NA**	**NA**	**NA**	**0.000**	**0.000**	Merkel, et al. 2017 [2]
**HLA-DPB1 rs1042169**	**G vs. A**	2.82 (2.54 – 3.13)	1.12E-84	*AAV*	*Caucasian*	*1986/4723*	NA	NA	**NA**	**NA**	**NA**	**NA**	**0.000**	**0.000**	Merkel, et al. 2017 [2]
**HLA-DPB1 rs141530233**	**A del**	2.99 (2.69 – 3.33)	1.13E-89	*AAV*	*Caucasian*	*1986/4723*	NA	NA	**NA**	**NA**	**NA**	**NA**	**0.000**	**0.000**	Merkel, et al. 2017 [2]
**HLA-DQA1 rs35242582**	**A vs. G**	1.60 (1.46 – 1.76)	6.34E-23	*AAV*	*Caucasian*	*1986/4723*	NA	NA	**NA**	**NA**	**NA**	**NA**	**0.000**	**0.000**	Merkel, et al. 2017 [2]
**HLA-DQB1 rs1049072**	**A vs. G**	1.40 (1.28 – 1.53)	6.46E-13	*AAV*	*Caucasian*	*1986/4723*	0.000	0.936	**0.000**	**0.000**	**0.000**	**0.000**	**0.000**	**0.000**	Merkel, et al. 2017 [2]
**PRTN3 rs62132293**	**G vs. C**	1.29 (1.19 – 1.39)	8.60E-11	*AAV*	*Caucasian*	*1986/4723*	0.029	1.000	**0.000**	**0.000**	**0.000**	**0.000**	**0.000**	**0.002**	Merkel, et al. 2017 [2]
**PTPN22 rs6679677**	**A vs. C**	1.40 (1.25 – 1.57)	1.88E-08	*AAV*	*Caucasian*	*1986/4723*	0.004	0.881	**0.002**	**0.172**	**0.000**	**0.001**	**0.001**	**0.447**	Merkel, et al. 2017 [2]

Gene/Variant	Comparison	OR (95% CI)	P-value	Diagnosis (clinical/serologic subtypes)	Ethnicity	No. of Cases/Controls	Power OR 1.2	Power OR 1.5	FPRP OR 1.2 0.001	FPRP OR 1.2 0.000001	FPRP OR 1.5 0.001	FPRP OR 1.5 0.000001	BFDP 0.001	BFDP 0.000001	Author, Year
**SERPINA1 rs28929474**	**T vs. C**	2.18 (1.75 – 2.71)	3.09E-12	*AAV*	*Caucasian*	*1986/4723*	0.000	0.000	**0.056**	0.855	**0.000**	**0.001**	**0.000**	**0.010**	Merkel, et al. 2017 [2]
**HLA-DPA1 rs9277341**	**C vs. T**	0.33 (0.28 – 0.39)	2.18E-39	*GPA*	*Caucasian*	*750/1820*	NA	NA	**NA**	**NA**	**NA**	**NA**	**0.000**	**0.000**	Xie, et al. 2013 [1]
**HLA-DPB1 rs9277554**	**T vs. C**	0.24 (0.20 – 0.30)	1.92E-50	*GPA*	*Caucasian*	*750/1820*	NA	NA	**NA**	**NA**	**NA**	**NA**	**0.000**	**0.000**	Xie, et al. 2013 [1]
**SEMA6A rs26595**	**C vs. T**	0.74 (0.67 – 0.82)	2.09E-08	*GPA*	*Caucasian*	*987/2731*	0.012	0.977	**0.001**	**0.071**	**0.000**	**0.001**	**0.001**	**0.423**	Xie, et al. 2013 [1]
**HLA-DQA2 rs3998159**	**C vs. A**	2.72 (2.24 – 3.22)	5.24E-25	*MPO-ANCA*	*Caucasian*	*378/4723*	NA	NA	**NA**	**NA**	**NA**	**NA**	**0.000**	**0.000**	Merkel, et al. 2017 [2]
**HLA-DQA2 rs7454108**	**C vs. T**	2.73 (2.25 – 3.24)	5.03E-25	*MPO-ANCA*	*Caucasian*	*378/4723*	NA	NA	**NA**	**NA**	**NA**	**NA**	**0.000**	**0.000**	Merkel, et al. 2017 [2]
**HLA-DQB1 rs1049072**	**A vs. G**	2.37 (2.01 – 2,78)	2.13E-24	*MPO-ANCA*	*Caucasian*	*378/4723*	NA	NA	**NA**	**NA**	**NA**	**NA**	**0.000**	**0.000**	Merkel, et al. 2017 [2]
**SNPs with statistically borderline significance (5.00E-08≤P<0.05)**															
**PTPN22(R620W) rs2476601**	**A vs. G**	1.36 (1.21 – 1.53)	1.86E-07	*AAV*	*Caucasian*	*1986/4723*	0.019	0.948	**0.016**	0.625	**0.000**	**0.032**	**0.020**	0.953	Merkel, et al. 2017 [2]
C3orf58 rs1512779	C vs. A	0.89 (0.79 – 0.99)	0.026	*GPA*	*Caucasian*	*987/2731*	0.887	1.000	0.973	1.000	0.970	1.000	0.998	1.000	Xie, et al. 2013 [1]
**CCDC86 rs595018**	**A vs. G**	1.46 (1.27 – 1.69)	1.60E-07	*GPA*	*Caucasian*	*1986/4723*	0.004	0.641	**0.084**	0.902	**0.001**	**0.058**	**0.033**	0.971	Xie, et al. 2013 [1]
**COBL rs1949829**	**T vs. C**	1.78 (1.42 – 2.24)	4.19E-07	*GPA*	*Caucasian*	*1986/4723*	0.000	0.072	0.694	0.996	**0.012**	0.549	**0.177**	0.995	Xie, et al. 2013 [1]
CTNNB1 rs9842536	T vs. C	1.17 (1.04 – 1.33)	9.70E-03	*GPA*	*Caucasian*	*987/2731*	0.651	1.000	0.962	1.000	0.942	0.999	0.996	1.000	Xie, et al. 2013 [1]
**DCTD rs4862110**	**C vs. T**	1.44 (1.24 – 1.67)	2.14E-06	*GPA*	*Caucasian*	*1986/4723*	0.008	0.705	**0.151**	0.947	**0.002**	**0.167**	**0.092**	0.990	Xie, et al. 2013 [1]
**DOK4 rs6023640**	**T vs. G**	1.29 (1.14 – 1.45)	2.73E-05	*GPA*	*Caucasian*	*987/2731*	0.113	0.994	**0.148**	0.946	**0.019**	0.664	**0.445**	0.999	Xie, et al. 2013 [1]
**FLJ34870 rs7585252**	**G vs. A**	1.26 (1.13 – 1.40)	1.74E-05	*GPA*	*Caucasian*	*987/2731*	0.182	0.999	**0.086**	0.904	**0.017**	0.632	**0.408**	0.999	Xie, et al. 2013 [1]
GRIA1 rs10515687	T vs. C	1.35 (1.15 – 1.59)	3.01E-04	*GPA*	*Caucasian*	*987/2731*	0.079	0.897	0.804	0.998	0.266	0.973	0.912	1.000	Xie, et al. 2013 [1]
OPCML rs4937787	C vs. A	0.82 (0.69 – 0.96)	0.016	*GPA*	*Caucasian*	*987/2731*	0.421	0.995	0.970	1.000	0.932	0.999	0.995	1.000	Xie, et al. 2013 [1]
**PAEP rs705669**	**G vs. A**	0.77 (0.68 – 0.87)	2.52E-05	*GPA*	*Caucasian*	*987/2731*	0.102	0.990	0.210	0.964	**0.027**	0.733	**0.520**	0.999	Xie, et al. 2013 [1]
RSPO4 rs6140836	C vs. T	0.75 (0.63 – 0.89)	7.09E-04	*GPA*	*Caucasian*	*987/2731*	0.114	0.911	0.896	0.999	0.519	0.991	0.962	1.000	Xie, et al. 2013 [1]
**WSCD1 rs7503953**	**A vs. C**	1.50 (1.29 – 1.76)	1.93E-07	*GPA*	*Caucasian*	*1986/4723*	0.003	0.500	**0.176**	0.955	**0.001**	**0.117**	**0.058**	0.984	Xie, et al. 2013 [1]
HLA-DQB1 rs1049072	A vs. G	1.17 (1.06 – 1.31)	3.82E-03	*PR3-ANCA*	*Caucasian*	*1361/4723*	0.670	1.000	0.906	0.999	0.866	0.998	0.992	1.000	Merkel, et al. 2017 [2]

Table S6. Re-analysis of the SNPs discovered in genome-wide association studies of patients with GPA. Xie, et al. only provided GPA SNPs with ORs and 95% CIs.

Gene/Variant	Comparison	OR (95% CI)	P-value	*Diagnosis (clinical/serologic subtypes)*	*Ethnicity*	*No. of Cases/Controls*	Power OR 1.2	Power OR 1.5	FPRP Values at Prior probability				BFDP 0.001	BFDP 0.000001	Author, Year
									OR 1.2		OR 1.5				
									0.001	0.000001	0.001	0.000001			
**SNPs with P-value <5.00E-08**															
**HLA-DOA rs3130604**	**G vs. A**	1.67 (1.39-2.02)	4.39E-08	*GPA*	*Caucasian*	*459/1503*	0.000	0.134	0.277	0.997	**0.001**	0.487	**0.025**	0.962	Xie, et al. 2013 [1]
**HLA-DOA rs763469**	**A vs. G**	1.70 (1.41-2.04)	1.46E-08	*GPA*	*Caucasian*	*459/1503*	0.000	0.089	**0.114**	0.992	**0.000**	**0.116**	**0.003**	**0.764**	Xie, et al. 2013 [1]
**HLA-DPA1 rs2395309**	**G vs. A**	0.27 (0.20-0.36)	2.15E-19	*GPA*	*Caucasian*	*459/1503*	NA	NA	**NA**	**NA**	**NA**	**NA**	**0.000**	**0.000**	Xie, et al. 2013 [1]
**HLA-DPA1 rs3077**	**C vs. T**	0.27 (0.20-0.36)	2.68E-19	*GPA*	*Caucasian*	*459/1503*	NA	NA	**NA**	**NA**	**NA**	**NA**	**0.000**	**0.000**	Xie, et al. 2013 [1]
**HLA-DPA1 rs2301226**	**T vs. C**	0.48 (0.36-0.62)	4.85E-08	*GPA*	*Caucasian*	*459/1503*	0.000	0.006	0.613	0.999	**0.003**	0.762	**0.023**	0.959	Xie, et al. 2013 [1]
**HLA-DPA1 rs9277341**	**C vs. T**	0.30 (0.25-0.38)	1.84E-30	*GPA*	*Caucasian*	*459/1503*	NA	NA	**NA**	**NA**	**NA**	**NA**	**0.000**	**0.000**	Xie, et al. 2013 [1]
**HLA-DPB1 rs987870**	**C vs. T**	0.26 (0.19-0.37)	6.09E-16	*GPA*	*Caucasian*	*459/1503*	0.000	0.000	0.597	0.999	**0.001**	0.462	**0.000**	**0.155**	Xie, et al. 2013 [1]
**HLA-DPB1 rs9277535**	**G vs. A**	0.24 (0.19-0.32)	2.12E-28	*GPA*	*Caucasian*	*459/1503*	NA	NA	**NA**	**NA**	**NA**	**NA**	**0.000**	**0.000**	Xie, et al. 2013 [1]
**HLA-DPB1 rs9277554**	**T vs. C**	0.22 (0.17-0.28)	4.88E-38	*GPA*	*Caucasian*	*459/1503*	NA	NA	**NA**	**NA**	**NA**	**NA**	**0.000**	**0.000**	Xie, et al. 2013 [1]
**HLA-DPB1 rs9277565**	**T vs. C**	0.24 (0.18-0.32)	1.91E-24	*GPA*	*Caucasian*	*459/1503*	NA	NA	**NA**	**NA**	**NA**	**NA**	**0.000**	**0.000**	Xie, et al. 2013 [1]
**HLA-DPB1 rs2281389**	**C vs. T**	0.24 (0.18-0.34)	1.69E-20	*GPA*	*Caucasian*	*459/1503*	0.000	0.000	0.458	0.999	**0.000**	**0.187**	**0.000**	**0.013**	Xie, et al. 2013 [1]
**HLA-DPB1 rs3128917**	**G vs. T**	0.22 (0.17-0.29)	4.92E-33	*GPA*	*Caucasian*	*459/1503*	NA	NA	**NA**	**NA**	**NA**	**NA**	**0.000**	**0.000**	Xie, et al. 2013 [1]
**HLA-DPB1 rs3117222**	**A vs. G**	0.22 (0.17-0.29)	3.05E-33	*GPA*	*Caucasian*	*459/1503*	NA	NA	**NA**	**NA**	**NA**	**NA**	**0.000**	**0.000**	Xie, et al. 2013 [1]
**HLA-DPB2 rs2064478**	**A vs. G**	0.22 (0.17-0.30)	4.29E-29	*GPA*	*Caucasian*	*459/1503*	NA	NA	**NA**	**NA**	**NA**	**NA**	**0.000**	**0.000**	Xie, et al. 2013 [1]
**HLA-DPB2 rs3130215**	**A vs. G**	2.42 (2.08-2.82)	2.37E-30	*GPA*	*Caucasian*	*459/1503*	NA	NA	**NA**	**NA**	**NA**	**NA**	**0.000**	**0.000**	Xie, et al. 2013 [1]
**HLA-DPB2 rs3117230**	**C vs. T**	0.22 (0.17-0.30)	4.29E-29	*GPA*	*Caucasian*	*459/1503*	NA	NA	**NA**	**NA**	**NA**	**NA**	**0.000**	**0.000**	Xie, et al. 2013 [1]
**HLA-DPB2 rs1883414**	**T vs. C**	0.53 (0.44-0.64)	1.13E-11	*GPA*	*Caucasian*	*459/1503*	0.000	0.009	**0.031**	0.970	**0.000**	**0.005**	**0.000**	**0.039**	Xie, et al. 2013 [1]
**HLA-DPB2 rs4713607**	**A vs. G**	0.60 (0.52-0.70)	6.70E-11	*GPA*	*Caucasian*	*459/1503*	0.000	0.090	**0.006**	0.849	**0.000**	**0.001**	**0.000**	**0.027**	Xie, et al. 2013 [1]
**HLA-DPB2 rs3129274**	**G vs. A**	1.56 (1.34-1.82)	1.35E-08	*GPA*	*Caucasian*	*459/1503*	0.000	0.309	**0.036**	0.974	**0.000**	**0.048**	**0.002**	**0.708**	Xie, et al. 2013 [1]
**HLA-DPB2 rs3117016**	**T vs. C**	0.48 (0.41-0.57)	1.09E-17	*GPA*	*Caucasian*	*459/1503*	NA	NA	**NA**	**NA**	**NA**	**NA**	**0.000**	**0.000**	Xie, et al. 2013 [1]
**HLA-DPB2 rs3117008**	**T vs. C**	0.60 (0.51-0.70)	4.90E-11	*GPA*	*Caucasian*	*459/1503*	0.000	0.090	**0.006**	0.849	**0.000**	**0.001**	**0.000**	**0.027**	Xie, et al. 2013 [1]
**HLA-DPB2 rs3117004**	**C vs. T**	0.57 (0.48-0.68)	1.90E-10	*GPA*	*Caucasian*	*459/1503*	0.000	0.041	**0.034**	0.972	**0.000**	**0.010**	**0.000**	**0.161**	Xie, et al. 2013 [1]
**HLA-DPB2 rs6901221**	**C vs. A**	0.42 (0.32-0.55)	6.08E-11	*GPA*	*Caucasian*	*459/1503*	0.000	0.000	0.475	0.999	**0.001**	0.423	**0.002**	**0.651**	Xie, et al. 2013 [1]
**COL11A2 rs986521**	**C vs. T**	1.85 (1.57-2.16)	2.91E-14	*GPA*	*Caucasian*	*459/1503*	0.000	0.004	**0.000**	0.246	**0.000**	**0.000**	**0.000**	**0.000**	Xie, et al. 2013 [1]
**COL11A2 rs2855430**	**T vs. C**	0.33 (0.24-0.45)	3.28E-13	*GPA*	*Caucasian*	*459/1503*	0.000	0.000	0.505	0.999	**0.001**	0.357	**0.000**	**0.237**	Xie, et al. 2013 [1]
**COL11A2 rs2855425**	**C vs. T**	1.80 (1.54-2.11)	7.77E-14	*GPA*	*Caucasian*	*459/1503*	0.000	0.012	**0.001**	0.594	**0.000**	**0.000**	**0.000**	**0.000**	Xie, et al. 2013 [1]
**COL11A2 rs2855459**	**T vs. C**	0.32 (0.23-0.44)	2.14E-13	*GPA*	*Caucasian*	*459/1503*	0.000	0.000	0.548	0.999	**0.001**	0.427	**0.000**	**0.287**	Xie, et al. 2013 [1]
**RXRB rs6531**	**C vs. T**	1.80 (1.54-2.11)	8.48E-14	*GPA*	*Caucasian*	*459/1503*	0.000	0.012	**0.001**	0.594	**0.000**	**0.000**	**0.000**	**0.000**	Xie, et al. 2013 [1]
**HSD17B8 rs439205**	**T vs. C**	0.31 (0.24-0.39)	3.51E-23	*GPA*	*Caucasian*	*459/1503*	NA	NA	**NA**	**NA**	**NA**	**NA**	**0.000**	**0.000**	Xie, et al. 2013 [1]
**HSD17B8 rs421446**	**C vs. T**	0.39 (0.31-0.48)	8.90E-20	*GPA*	*Caucasian*	*459/1503*	NA	NA	**NA**	**NA**	**NA**	**NA**	**0.000**	**0.000**	Xie, et al. 2013 [1]
**RING1 rs213213**	**A vs. G**	1.83 (1.57-2.14)	6.98E-15	*GPA*	*Caucasian*	*459/1503*	0.000	0.006	**0.001**	0.375	**0.000**	**0.000**	**0.000**	**0.000**	Xie, et al. 2013 [1]

**RING1 rs213212**	**G vs. T**	1.85 (1.58-2.17)	7.63E-15	*GPA*	*Caucasian*	*459/1503*	0.000	0.005	**0.001**	0.439	**0.000**	**0.000**	**0.000**	**0.000**	Xie, et al. 2013 [1]
**COBL rs1949829**	**T vs. C**	2.19 (1.68-2.86)	3.58E-09	*GPA*	*Caucasian*	*459/1503*	0.000	0.003	0.632	0.999	**0.003**	0.759	**0.017**	0.946	Xie, et al. 2013 [1]
**CCDC86 rs595018**	**A vs. G**	1.61 (1.36-1.90)	2.74E-08	*GPA*	*Caucasian*	*459/1503*	0.000	0.201	**0.064**	0.986	**0.000**	**0.080**	**0.003**	**0.764**	Xie, et al. 2013 [1]
**WSCD1 rs7503953**	**A vs. C**	1.72 (1.44-2.06)	1.39E-09	*GPA*	*Caucasian*	*459/1503*	0.000	0.068	**0.076**	0.988	**0.000**	**0.053**	**0.001**	**0.555**	Xie, et al. 2013 [1]
**SNPs reported non-significant (5.00E-08≤P<1.00E-04)**															
**TCEB3 rs2076346**	**C vs. T**	1.46 (1.24-1.73)	8.62E-06	*GPA*	*Caucasian*	*459/1503*	0.012	0.623	0.512	0.999	**0.019**	0.952	**0.433**	0.999	Xie, et al. 2013 [1]
**DAB1 rs264036**	**C vs. T**	0.72 (0.62-0.85)	9.82E-05	*GPA*	*Caucasian*	*459/1503*	0.042	0.818	0.713	1.000	**0.113**	0.992	**0.807**	1.000	Xie, et al. 2013 [1]
**DAB1 rs542873**	**T vs. C**	1.37 (1.18-1.59)	4.70E-05	*GPA*	*Caucasian*	*459/1503*	0.041	0.884	0.457	0.999	**0.037**	0.975	**0.601**	0.999	Xie, et al. 2013 [1]
**DAB1 rs197644**	**G vs. A**	1.37 (1.18-1.59)	3.89E-05	*GPA*	*Caucasian*	*459/1503*	0.041	0.884	0.457	0.999	**0.037**	0.975	**0.601**	0.999	Xie, et al. 2013 [1]
**LPHN2 rs11579502**	**C vs. T**	1.56 (1.28-1.90)	9.43E-06	*GPA*	*Caucasian*	*459/1503*	0.005	0.348	0.684	1.000	**0.027**	0.966	**0.458**	0.999	Xie, et al. 2013 [1]
SCYL1BP1 rs16863343	C vs. T	2.18 (1.46-3.24)	8.15E-05	*GPA*	*Caucasian*	*459/1503*	0.002	0.032	0.987	1.000	0.782	1.000	0.970	1.000	Xie, et al. 2013 [1]
**NCKAP5 rs1134119**	**C vs. T**	1.79 (1.38-2.32)	8.96E-06	*GPA*	*Caucasian*	*459/1503*	0.001	0.091	0.896	1.000	**0.106**	0.992	**0.658**	0.999	Xie, et al. 2013 [1]
**NCKAP5 rs7585252**	**G vs. A**	1.35 (1.16-1.57)	9.69E-05	*GPA*	*Caucasian*	*459/1503*	0.063	0.914	0.607	0.999	**0.096**	0.991	**0.785**	1.000	Xie, et al. 2013 [1]
**NEK10 rs1579900**	**T vs. G**	1.51 (1.24-1.85)	3.97E-05	*GPA*	*Caucasian*	*459/1503*	0.013	0.474	0.840	1.000	**0.128**	0.993	0.800	1.000	Xie, et al. 2013 [1]
**CTNNB1 rs9842536**	**T vs. C**	1.46 (1.23-1.74)	1.63E-05	*GPA*	*Caucasian*	*459/1503*	0.014	0.619	0.624	0.999	**0.037**	0.974	**0.575**	0.999	Xie, et al. 2013 [1]
**C3orf58 rs1512779**	**C vs. A**	0.72 (0.61-0.84)	2.86E-05	*GPA*	*Caucasian*	*459/1503*	0.032	0.836	0.484	0.999	**0.034**	0.972	**0.579**	0.999	Xie, et al. 2013 [1]
**PLSCR4 rs7628805**	**A vs. C**	1.41 (1.18-1.67)	9.72E-05	*GPA*	*Caucasian*	*459/1503*	0.031	0.763	0.691	1.000	**0.083**	0.989	**0.754**	1.000	Xie, et al. 2013 [1]
**ST6GAL1 rs10513807**	**G vs. A**	0.71 (0.61-0.83)	9.49E-06	*GPA*	*Caucasian*	*459/1503*	0.022	0.785	0.436	0.999	**0.021**	0.956	**0.471**	0.999	Xie, et al. 2013 [1]
**KIAA0746 rs4269167**	**T vs. C**	0.73 (0.63-0.86)	7.72E-05	*GPA*	*Caucasian*	*459/1503*	0.057	0.861	0.747	1.000	**0.163**	0.995	0.858	1.000	Xie, et al. 2013 [1]
**DCTD rs4862110**	**C vs. T**	1.63 (1.36-1.94)	5.00E-08	*GPA*	*Caucasian*	*459/1503*	0.000	0.175	**0.118**	0.993	**0.000**	**0.178**	**0.007**	0.878	Xie, et al. 2013 [1]
**OSMR rs357291**	**C vs. A**	0.72 (0.62-0.84)	2.81E-05	*GPA*	*Caucasian*	*459/1503*	0.032	0.836	0.484	0.999	**0.034**	0.972	**0.579**	0.999	Xie, et al. 2013 [1]
**SEMA6A rs26595**	**C vs. T**	0.74 (0.63-0.86)	9.58E-05	*GPA*	*Caucasian*	*459/1503*	0.061	0.913	0.586	0.999	**0.086**	0.989	**0.766**	1.000	Xie, et al. 2013 [1]
**GRIA1 rs10515687**	**T vs. C**	1.56 (1.25-1.94)	8.11E-05	*GPA*	*Caucasian*	*459/1503*	0.009	0.362	0.874	1.000	**0.150**	0.994	0.811	1.000	Xie, et al. 2013 [1]
**WWC1 rs3853242**	**G vs. A**	0.74 (0.63-0.86)	8.55E-05	*GPA*	*Caucasian*	*459/1503*	0.061	0.913	0.586	0.999	**0.086**	0.989	**0.766**	1.000	Xie, et al. 2013 [1]
**ERGIC1 rs1564259**	**A vs. G**	0.69 (0.58-0.83)	7.19E-05	*GPA*	*Caucasian*	*459/1503*	0.023	0.642	0.785	1.000	**0.114**	0.992	**0.797**	1.000	Xie, et al. 2013 [1]
**ERGIC1 rs1006721**	**C vs. T**	0.69 (0.58-0.83)	6.22E-05	*GPA*	*Caucasian*	*459/1503*	0.023	0.642	0.785	1.000	**0.114**	0.992	**0.797**	1.000	Xie, et al. 2013 [1]
**OFCC1 rs9358619**	**A vs. G**	1.45 (1.21-1.74)	4.90E-05	*GPA*	*Caucasian*	*459/1503*	0.021	0.642	0.756	1.000	**0.092**	0.990	**0.762**	1.000	Xie, et al. 2013 [1]
**HLA-DMA rs3135029**	**A vs. C**	1.61 (1.28-2.02)	4.55E-05	*GPA*	*Caucasian*	*459/1503*	0.006	0.270	0.875	1.000	**0.125**	0.993	**0.764**	1.000	Xie, et al. 2013 [1]
BRD2 rs3130597	T vs. C	1.60 (1.28-2.02)	4.80E-05	*GPA*	*Caucasian*	*459/1503*	0.008	0.294	0.909	1.000	0.209	0.996	0.850	1.000	Xie, et al. 2013 [1]
**HLA-DOA rs176248**	**T vs. C**	0.70 (0.58-0.83)	8.16E-05	*GPA*	*Caucasian*	*459/1503*	0.022	0.713	0.644	0.999	**0.054**	0.983	**0.668**	1.000	Xie, et al. 2013 [1]
**HLA-DOA rs206762**	**C vs. T**	1.36 (1.17-1.58)	5.92E-05	*GPA*	*Caucasian*	*459/1503*	0.051	0.900	0.534	0.999	**0.061**	0.985	**0.703**	1.000	Xie, et al. 2013 [1]
HLA-DOA rs429916	A vs. C	0.37 (0.25-0.56)	4.11E-07	*GPA*	*Caucasian*	*459/1503*	0.000	0.003	0.977	1.000	0.490	0.999	0.862	1.000	Xie, et al. 2013 [1]
**HLA-DOA rs9296068**	**G vs. T**	0.68 (0.58-0.81)	7.49E-06	*GPA*	*Caucasian*	*459/1503*	0.011	0.588	0.578	0.999	**0.026**	0.964	**0.491**	0.999	Xie, et al. 2013 [1]
HLA-DPB1 rs3130192	T vs. C	1.60 (1.27-2.02)	7.51E-05	*GPA*	*Caucasian*	*459/1503*	0.008	0.294	0.909	1.000	0.209	0.996	0.850	1.000	Xie, et al. 2013 [1]
**HLA-DPB2 rs1810472**	**G vs. A**	0.65 (0.54-0.77)	1.06E-06	*GPA*	*Caucasian*	*459/1503*	0.002	0.385	0.235	0.997	**0.002**	0.618	**0.062**	0.985	Xie, et al. 2013 [1]
**HLA-DPB2 rs3117035**	**A vs. G**	0.72 (0.61-0.84)	2.50E-05	*GPA*	*Caucasian*	*459/1503*	0.032	0.836	0.484	0.999	**0.034**	0.972	**0.579**	0.999	Xie, et al. 2013 [1]
**COL11A2 rs2235498**	**T vs. C**	0.67 (0.55-0.81)	3.86E-05	*GPA*	*Caucasian*	*459/1503*	0.012	0.521	0.744	1.000	**0.063**	0.985	**0.678**	1.000	Xie, et al. 2013 [1]
COL11A2 rs9368758	A vs. G	0.44 (0.30-0.65)	3.28E-05	*GPA*	*Caucasian*	*459/1503*	0.001	0.018	0.982	1.000	0.669	1.000	0.946	1.000	Xie, et al. 2013 [1]
COL11A2 rs2269346	A vs. G	0.44 (0.30-0.66)	3.64E-05	*GPA*	*Caucasian*	*459/1503*	0.001	0.022	0.986	1.000	0.764	1.000	0.965	1.000	Xie, et al. 2013 [1]
**WDR46 rs3130257**	**T vs. C**	1.63 (1.32-2.01)	5.61E-06	*GPA*	*Caucasian*	*459/1503*	0.002	0.218	0.700	1.000	**0.022**	0.957	**0.365**	0.998	Xie, et al. 2013 [1]
**DAXX rs211474**	**T vs. C**	0.71 (0.60-0.84)	6.37E-05	*GPA*	*Caucasian*	*459/1503*	0.031	0.769	0.679	1.000	**0.078**	0.988	**0.744**	1.000	Xie, et al. 2013 [1]
**KIFC1 rs211452**	**C vs. T**	0.65 (0.55-0.76)	1.98E-07	*GPA*	*Caucasian*	*459/1503*	0.001	0.375	**0.067**	0.986	**0.000**	**0.150**	**0.008**	0.895	Xie, et al. 2013 [1]
**SYNGAP1 rs211456**	**A vs. C**	0.72 (0.61-0.84)	3.57E-05	*GPA*	*Caucasian*	*459/1503*	0.032	0.836	0.484	0.999	**0.034**	0.972	**0.579**	0.999	Xie, et al. 2013 [1]
**SYNGAP1 rs2247385**	**G vs. A**	0.69 (0.59-0.81)	5.14E-06	*GPA*	*Caucasian*	*459/1503*	0.011	0.663	0.353	0.998	**0.009**	0.896	**0.271**	0.997	Xie, et al. 2013 [1]
**FLJ43752 rs210120**	**G vs. A**	0.72 (0.62-0.84)	2.58E-05	*GPA*	*Caucasian*	*459/1503*	0.032	0.836	0.484	0.999	**0.034**	0.972	**0.579**	0.999	Xie, et al. 2013 [1]
**BCKDHB rs515347**	**G vs. A**	1.70 (1.32-2.18)	3.13E-05	*GPA*	*Caucasian*	*459/1503*	0.003	0.162	0.905	1.000	**0.151**	0.994	**0.769**	1.000	Xie, et al. 2013 [1]
**TCBA1 rs6924068**	**G vs. A**	1.40 (1.20-1.63)	1.37E-05	*GPA*	*Caucasian*	*459/1503*	0.023	0.813	0.382	0.998	**0.018**	0.947	**0.429**	0.999	Xie, et al. 2013 [1]
**MAGI2 rs3779312**	**A vs. G**	1.50 (1.26-1.78)	5.26E-06	*GPA*	*Caucasian*	*459/1503*	0.005	0.500	0.392	0.998	**0.007**	0.873	**0.214**	0.996	Xie, et al. 2013 [1]
**CUTL1 rs1734729**	**T vs. C**	1.41 (1.20-1.67)	4.54E-05	*GPA*	*Caucasian*	*459/1503*	0.031	0.763	0.691	1.000	**0.083**	0.989	**0.754**	1.000	Xie, et al. 2013 [1]
**DPP6 rs4726422**	**G vs. A**	0.74 (0.63-0.86)	7.74E-05	*GPA*	*Caucasian*	*459/1503*	0.061	0.913	0.586	0.999	**0.086**	0.989	**0.766**	1.000	Xie, et al. 2013 [1]
**LOC441376 rs3019885**	**G vs. T**	1.44 (1.24-1.67)	1.90E-06	*GPA*	*Caucasian*	*459/1503*	0.008	0.705	**0.151**	0.994	**0.002**	0.667	**0.092**	0.990	Xie, et al. 2013 [1]
**SLC30A8 rs1793729**	**C vs. T**	0.68 (0.57-0.80)	5.68E-06	*GPA*	*Caucasian*	*459/1503*	0.007	0.594	0.317	0.998	**0.006**	0.847	**0.194**	0.996	Xie, et al. 2013 [1]
**SLC30A8 rs1695715**	**T vs. C**	0.69 (0.58-0.82)	1.57E-05	*GPA*	*Caucasian*	*459/1503*	0.016	0.652	0.610	0.999	**0.037**	0.975	**0.582**	0.999	Xie, et al. 2013 [1]
**KCNK9 rs2447406**	**T vs. C**	1.58 (1.27-1.97)	3.25E-05	*GPA*	*Caucasian*	*459/1503*	0.007	0.322	0.869	1.000	**0.130**	0.993	**0.781**	1.000	Xie, et al. 2013 [1]
**C9orf66 rs584922**	**T vs. C**	0.70 (0.60-0.82)	1.30E-05	*GPA*	*Caucasian*	*459/1503*	0.015	0.727	0.392	0.998	**0.013**	0.932	**0.365**	0.998	Xie, et al. 2013 [1]
**C9orf93 rs1341740**	**T vs. C**	1.52 (1.23-1.87)	9.73E-05	*GPA*	*Caucasian*	*459/1503*	0.013	0.450	0.855	1.000	**0.142**	0.994	0.814	1.000	Xie, et al. 2013 [1]
**LRRN6C rs10491888**	**G vs. A**	1.54 (1.25-1.91)	5.75E-05	*GPA*	*Caucasian*	*459/1503*	0.012	0.405	0.880	1.000	**0.173**	0.995	0.837	1.000	Xie, et al. 2013 [1]
**PAEP rs705669**	**G vs. A**	0.69 (0.57-0.83)	8.53E-05	*GPA*	*Caucasian*	*459/1503*	0.023	0.642	0.785	1.000	**0.114**	0.992	**0.797**	1.000	Xie, et al. 2013 [1]
**NEUROG3 rs731573**	**T vs. C**	1.44 (1.20-1.72)	9.40E-05	*GPA*	*Caucasian*	*459/1503*	0.022	0.674	0.722	1.000	**0.079**	0.988	**0.738**	1.000	Xie, et al. 2013 [1]
PTEN rs7084813	A vs. G	0.59 (0.46-0.76)	2.79E-05	*GPA*	*Caucasian*	*459/1503*	0.004	0.172	0.922	1.000	0.204	0.996	0.824	1.000	Xie, et al. 2013 [1]
OPCML rs4937787	C vs. A	0.60 (0.46-0.77)	5.75E-05	*GPA*	*Caucasian*	*459/1503*	0.005	0.204	0.924	1.000	0.227	0.997	0.847	1.000	Xie, et al. 2013 [1]
**TMPO rs2216021**	**C vs. T**	1.36 (1.17-1.57)	6.14E-05	*GPA*	*Caucasian*	*459/1503*	0.044	0.909	0.382	0.998	**0.029**	0.967	**0.544**	0.999	Xie, et al. 2013 [1]
**TMPO rs2011247**	**C vs. T**	0.73 (0.63-0.85)	4.84E-05	*GPA*	*Caucasian*	*459/1503*	0.044	0.879	0.534	0.999	**0.054**	0.983	**0.680**	1.000	Xie, et al. 2013 [1]
**FGF9 rs2031421**	**T vs. G**	1.44 (1.21-1.72)	4.01E-05	*GPA*	*Caucasian*	*459/1503*	0.022	0.674	0.722	1.000	**0.079**	0.988	**0.738**	1.000	Xie, et al. 2013 [1]

RSPO4 rs6140836	C vs. T	0.58 (0.44-0.76)	7.26E-05	*GPA*	*Caucasian*	*459/1503*	0.004	0.156	0.948	1.000	0.333	0.998	0.890	1.000	Xie, et al. 2013 [1]
**DOK5 rs6023640**	**T vs. G**	1.40 (1.18-1.66)	8.98E-05	*GPA*	*Caucasian*	*459/1503*	0.038	0.786	0.740	1.000	**0.121**	0.993	0.815	1.000	Xie, et al. 2013 [1]
**PDE9A rs2269127**	**A vs. G**	1.65 (1.36-2.01)	4.06E-07	*GPA*	*Caucasian*	*459/1503*	0.001	0.172	0.457	0.999	**0.004**	0.793	**0.094**	0.990	Xie, et al. 2013 [1]

Table S7. These gene sets were computed in gene ontology enrichment analysis and protein-protein interaction network analysis. This gene set includes noteworthy variants from meta-analyses and statistically significant GWAS SNPs.

From :

- Noteworthy variants from [Association of granulomatosis with polyangiitis (Wegener's) with HLA-DPB1*04 and SEMA6A gene variants: evidence from genome-wide analysis, Xie G, et al. 2013. Supplementary data (GPA)]
- Statistically significant GWAS SNPs from [Identification of Functional and Expression Polymorphisms Associated With Risk for Antineutrophil Cytoplasmic Autoantibody-Associated Vasculitis, Merkel PA, et al. 2017]
- Noteworthy variants from all the included meta-analyses studies in this review

ANCA-associated vasculitis		Granulomatous polyangiitis (GPA)		PR3-ANCA		MPO-ANCA	
**Gene**	**SNP ID**	**Gene**	**SNP ID**	**Gene**	**SNP ID**	**Gene**	**SNP ID**
AGER	rs2070600	BCKDHB	rs515347	BRD2	rs206762	BTNL2	rs3793127
CD226	rs763361	C3orf58	rs1512779	COL11A2	Affx-52341735	C6orf10	rs6910071
CFB	rs541862	C9orf66	rs584922	COL11A2	rs2071025	HLA-DQA2	rs3998159
COL11A2	Affx-52341735	C9orf93	rs1341740	COL11A2	rs2254287	HLA-DQA2	rs7454108
COL11A2	rs2076310	CCDC86	rs595018	COL11A2	rs2257126	HLA-DQA2	rs9275184
COL11A2	rs2254287	CD226	rs763361	COL11A2	rs2294479	HLA-DQB1	rs1049072
COL11A2	rs2257126	CTLA-4	rs3087243	COL11A2	rs2744512	HLA-DQB1	rs1130399

COL11A2	rs2294479	COBL	rs1949829	COL11A2	rs2855425	HLA-DRA	rs2395163
COL11A2	rs2855425	COL11A2	rs2235498	COL11A2	rs2855437	HLA-DRA	rs2395175
COL11A2	rs2855437	COL11A2	rs2855425	COL11A2	rs2855442	HLA-DRB1	rs28366302
COL11A2	rs2855442	COL11A2	rs2855430	COL11A2	rs2855448	HLA-DRB1	rs34252386
COL11A2	rs2855448	COL11A2	rs2855459	COL11A2	rs2855459	HLA-DRB1	rs35445101
COL11A2	rs2855459	COL11A2	rs986521	COL11A2	rs3116994	HLA-DRB1	rs660895
COL11A2	rs3116994	CTLA-4	$(AT)_{86}$	COL11A2	rs3116999	HLA-DRB1	rs9271824
COL11A2	rs3116999	CTNNB1	rs9842536	COL11A2	rs3129207	HLA-DRB1	rs9271897
COL11A2	rs3129207	CUTL1	rs1734729	COL11A2	rs3129234	HLA-DRB1	rs9272105
COL11A2	rs3129267	DAB1	rs197644	COL11A2	rs3129252	HLA-DRB5	rs2395185
COL11A2	rs3129270	DAB1	rs264036	COL11A2	rs3129258	HLA-DRB5	rs9268923
COL11A2	rs3130161	DAB1	rs542873	COL11A2	rs3129264	OR2W1	rs34892006
COL11A2	rs726599	DAXX	rs211474	COL11A2	rs3129267	TAP2	rs115360810
COL11A2	rs9277935	DCTD	rs4862110	COL11A2	rs3129270		
COL11A2	rs9368758	DOK4	rs6023640	COL11A2	rs3130161		
COL11A2	rs986521	DPP6	rs4726422	COL11A2	rs726599		
CTLA-4	$(AT)_{86}$	ERGIC1	rs1006721	COL11A2	rs756440		
CTLA-4	rs3087243 (CT60)	ERGIC1	rs1564259	COL11A2	rs9277935		

HLA-DOA	rs17214533	FGF9	rs2031421	COL11A2	rs9368758
HLA-DOA	rs2858458	FLJ34870 (NCKAP5)	rs7585252	COL11A2	rs986521
HLA-DOA	rs3097669	FLJ43752 (LINC00336)	rs210120	DAXX	rs211449
HLA-DOA	rs3129304	GRIA1	rs10515687	EHMT2	rs486416
HLA-DOA	rs3130176	HLA-DMA	rs3135029	EHMT2	rs605203
HLA-DOA	rs3130604	HLA-DOA	rs176248	HLA-C	rs2524040
HLA-DOA	rs6936620	HLA-DOA	rs206762	HLA-C	rs3873386
HLA-DOA	rs7634469	HLA-DOA	rs3130604	HLA-C	rs9468925
HLA-DOA	rs9296068	HLA-DOA	rs763469	HLA-DOA	rs17214533
HLA-DPA1	Affx-28512796	HLA-DOA	rs9296068	HLA-DOA	rs2858458
HLA-DPA1	Affx-28512827	HLA-DPA1	rs2301226	HLA-DOA	rs3097648
HLA-DPA1	rs10214910	HLA-DPA1	rs2395309	HLA-DOA	rs3097669
HLA-DPA1	rs1042190	HLA-DPA1	rs3077	HLA-DOA	rs3129304
HLA-DPA1	rs1042434	HLA-DPA1	rs9277341	HLA-DOA	rs3130176
HLA-DPA1	rs1126543	HLA-DPB1	rs2281389	HLA-DOA	rs3130604
HLA-DPA1	rs1126769	HLA-DPB1	rs3117222	HLA-DOA	rs423639
HLA-DPA1	rs1431399	HLA-DPB1	rs3128917	HLA-DOA	rs429916
HLA-DPA1	rs2308911	HLA-DPB1	rs9277535	HLA-DOA	rs763469

HLA-DPA1	rs3077	HLA-DPB1	rs9277554	HLA-DPA1	Affx-28512796
HLA-DPA1	rs9277341	HLA-DPB1	rs9277565	HLA-DPA1	Affx-28512827
HLA-DPA1	rs987870	HLA-DPB1	rs987870	HLA-DPA1	rs10214910
HLA-DPB1	rs1042151	HLA-DPB2	rs1810472	HLA-DPA1	rs1042190
HLA-DPB1	rs1042153	HLA-DPB2	rs1883414	HLA-DPA1	rs1042434
HLA-DPB1	rs1042169	HLA-DPB2	rs2064478	HLA-DPA1	rs1126543
HLA-DPB1	rs1042335	HLA-DPB2	rs3117004	HLA-DPA1	rs1126769
HLA-DPB1	rs1071597	HLA-DPB2	rs3117008	HLA-DPA1	rs1431399
HLA-DPB1	rs1126513	HLA-DPB2	rs3117016	HLA-DPA1	rs2308911
HLA-DPB1	rs141530233	HLA-DPB2	rs3117035	HLA-DPA1	rs3077
HLA-DPB1	rs1431403	HLA-DPB2	rs3117230	HLA-DPA1	rs9277341
HLA-DPB1	rs2064474	HLA-DPB2	rs3129274	HLA-DPA1	rs987870
HLA-DPB1	rs2064476	HLA-DPB2	rs3130215	HLA-DPB1	Affx-28513472
HLA-DPB1	rs2064478	HLA-DPB2	rs4713607	HLA-DPB1	rs1042151
HLA-DPB1	rs2068204	HLA-DPB2	rs6901221	HLA-DPB1	rs1042153
HLA-DPB1	rs2071352	HSD17B8	rs421446	HLA-DPB1	rs1042169
HLA-DPB1	rs2144014	HSD17B8	rs439205	HLA-DPB1	rs1042335
HLA-DPB1	rs2179920	IRF5	rs10954213	HLA-DPB1	rs1071597

HLA-DPB1	rs2281389	KCNK9	rs2447406	HLA-DPB1	rs1126513
HLA-DPB1	rs2395314	KIAA0746	rs4269167	HLA-DPB1	rs141530233
HLA-DPB1	rs3097671	KIFC1	rs211452	HLA-DPB1	rs1431403
HLA-DPB1	rs3117223	LOC441376	rs3019885	HLA-DPB1	rs2064474
HLA-DPB1	rs3117230	LPHN2	rs11579502	HLA-DPB1	rs2064476
HLA-DPB1	rs3117231	LRRN6C	rs10491888	HLA-DPB1	rs2064478
HLA-DPB1	rs3128917	MAGI2	rs3779312	HLA-DPB1	rs2068204
HLA-DPB1	rs3128927	NCKAP5	rs1134119	HLA-DPB1	rs2071352
HLA-DPB1	rs3130190	NCKAP5	rs7585252	HLA-DPB1	rs2144014
HLA-DPB1	rs31328921	NEK10	rs1579900	HLA-DPB1	rs2179920
HLA-DPB1	rs3135024	NEUROG3	rs731573	HLA-DPB1	rs2281389
HLA-DPB1	rs7772134	OFCC1	rs9358619	HLA-DPB1	rs2281390
HLA-DPB1	rs910320	OSMR	rs357291	HLA-DPB1	rs2395314
HLA-DPB1	rs9271897	PAEP	rs705669	HLA-DPB1	rs3097671
HLA-DPB1	rs9277341	PDE9A	rs2269127	HLA-DPB1	rs3117223
HLA-DPB1	rs9277410	PLSCR4	rs7628805	HLA-DPB1	rs3117230
HLA-DPB1	rs9277424	PTPN22	rs2476601	HLA-DPB1	rs3117231
HLA-DPB1	rs9277464	RING1	rs213212	HLA-DPB1	rs3128917

HLA-DPB1	rs9277471	RING1	rs213213	HLA-DPB1	rs3128921
HLA-DPB1	rs9277489	RXRB	rs6531	HLA-DPB1	rs3128927
HLA-DPB1	rs9277498	RXRB	rs9277935	HLA-DPB1	rs3130190
HLA-DPB1	rs9277514	SEMA6A	rs26595	HLA-DPB1	rs3135024
HLA-DPB1	rs9277535	SERPINA 1	Z allele	HLA-DPB1	rs7772134
HLA-DPB1	rs9277546	SLC30A8	rs1695715	HLA-DPB1	rs910320
HLA-DPB1	rs9277554	SLC30A8	rs1793729	HLA-DPB1	rs9277410
HLA-DPB1	rs9277567	ST6GAL1	rs10513807	HLA-DPB1	rs9277424
HLA-DPB2	rs1810472	SYNGAP1	rs211456	HLA-DPB1	rs9277464
HLA-DPB2	rs1883414	SYNGAP1	rs2247385	HLA-DPB1	rs9277471
HLA-DPB2	rs3117008	TCBA1	rs6924068	HLA-DPB1	rs9277489
HLA-DPB2	rs3117016	TCEB3	rs2076346	HLA-DPB1	rs9277498
HLA-DPB2	rs3129274	TMPO	rs2011247	HLA-DPB1	rs9277514
HLA-DPB2	rs3129294	TMPO	rs2216021	HLA-DPB1	rs9277535
HLA-DPB2	rs3130215	WDR46	rs3130257	HLA-DPB1	rs9277546
HLA-DQA1	rs17612576	WSCD1	rs7503953	HLA-DPB1	rs9277554
HLA-DQA1	rs17843619	WWC1	rs3853242	HLA-DPB1	rs9277567
HLA-DQA1	rs199556640			HLA-DPB1	rs9380343

HLA-DQA1	rs28584179
HLA-DQA1	rs35242582
HLA-DQA1	rs9272116
HLA-DQA1	rs9272346
HLA-DQA1	rs9273088
HLA-DQA1	rs9273215
HLA-DQB1	rs1049072
HLA-DQB1	rs1063355
HLA-DQB1	rs1130399
HLA-DQB1	rs9274552
HLA-DRA	rs2395175
HLA-DRB1	rs116518618
HLA-DRB1	rs660895
HLA-DRB1	rs9271824
HLA-DRB1	rs9272105
HLA-DRB4	-
HSD17B8	rs421446
HSD17B8	rs459205

HLA-DPB1*0401	
HLA-DPB2	rs1810472
HLA-DPB2	rs1883414
HLA-DPB2	rs3117008
HLA-DPB2	rs3117016
HLA-DPB2	rs3117034
HLA-DPB2	rs3129274
HLA-DPB2	rs3129294
HLA-DPB2	rs3130215
HLA-DQA1	rs28584179
HLA-DQA1	rs35242582
HLA-DRB1	rs116518618
HLA-DRB1*15	
HSD17B8	rs421446
HSD17B8	rs439205
MICA	rs1882
MICA	rs2596530
MUC22	rs3094672

IRF5	rs10954213
MCCD1	rs3132454
MICA	rs1882
MICA	rs2256183
MICA	rs2596530
MICA	rs75549913
NOTCH4	rs3096702
NOTCH4	rs3134926
NOTCH4	rs382259
PRTN3	rs62132293
PTPN22	(R620W) rs2476601
PTPN22	rs6679677
RING1	rs213194
RING1	rs213209
RING1	rs213210
RING1	rs213212
RING1	rs213213
RING1	rs439121

PRTN3	rs62132293
RING1	rs213194
RING1	rs213209
RING1	rs213210
RING1	rs213212
RING1	rs213213
RING1	rs439121
RING1	rs9277946
RXRB	rs2076310
RXRB	rs6531
RXRB	rs9277935
SERPINA 1	Z allele
SERPINA1	rs28929474
TLR9	rs352140
TLR9	rs352162
WDR46	rs3130257

RING1	rs9277946
RING1/RXRB	rs213213
RXRB	rs2076310
RXRB	rs6531
RXRB	rs9277935
SERPINA 1	Z allele
SERPINA1	rs28929474
SKIV2L	rs429608
SKIV2L	rs43899
TLR9	rs352162

Figure S1. The Venn diagram including all patients with AAV and those with GPA and the respective target antigens with the noteworthy SNPs from our FPRP and BFDP analyses.

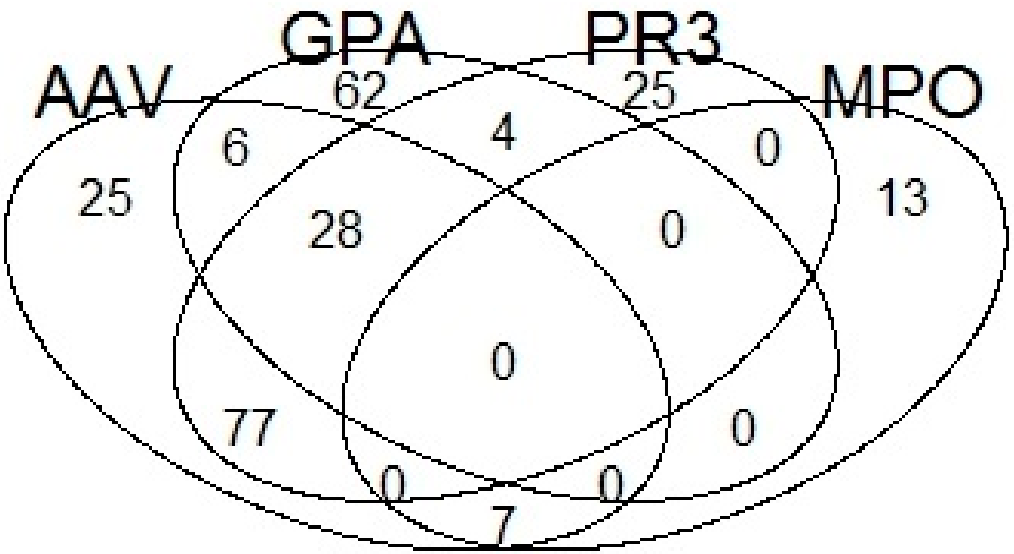

Figure S2. Protein–protein interaction network of GPA (A) and ANCA-associated vasculitis (B). Genes are weighted according to the number of SNPs of each gene. The gene set comprises the GWAS discovered SNP genes as well as noteworthy SNP genes from meta-analyses of observational studies and GWAS meta-analyses. Borderline SNPs ($5.0\times10^{-8} < p < 0.05$) from GWASs that were noteworthy in our analysis were also included. The yellow nodes represent the genes associated with interferon-gamma-mediated signaling pathway.

A)

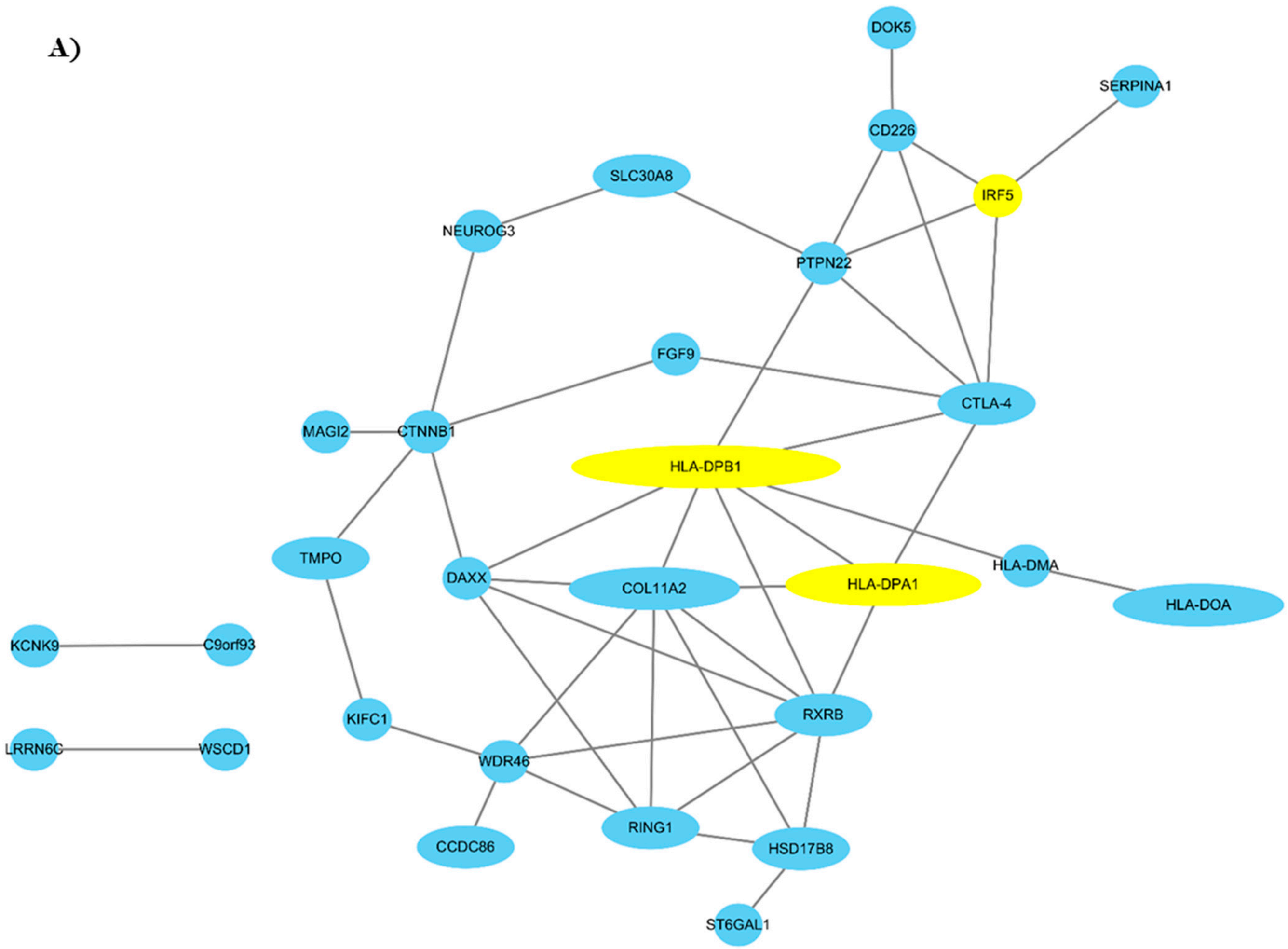

B)

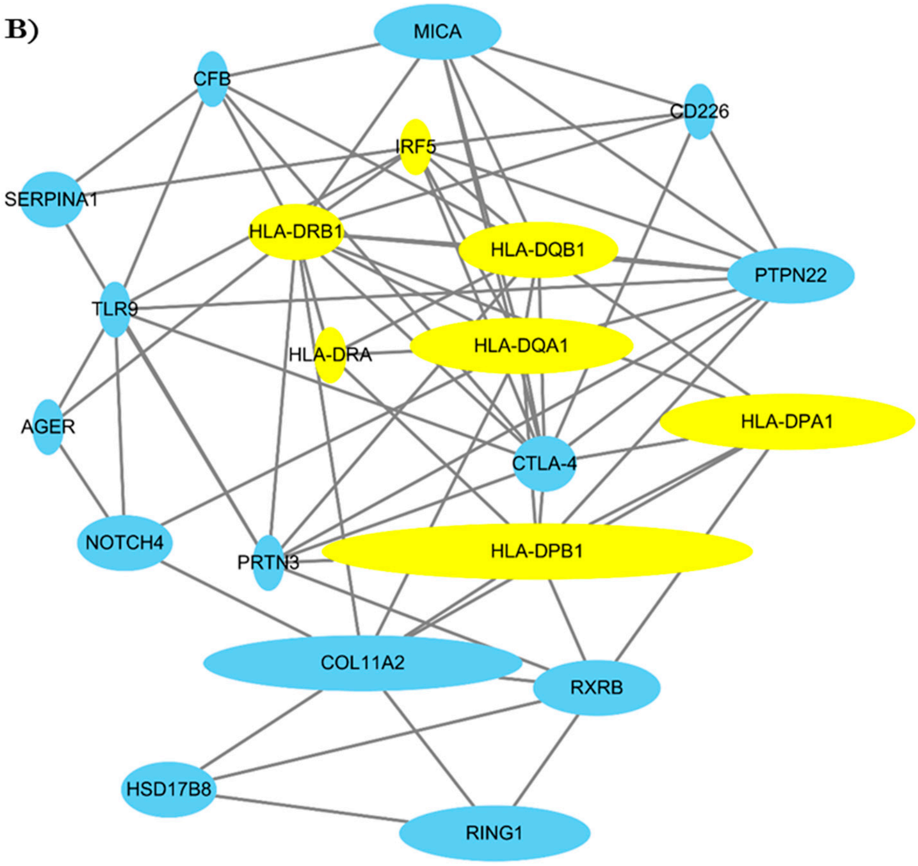
MICA
CFB
CD226
IRF5
SERPINA1
HLA-DRB1
HLA-DQB1
PTPN22
TLR9
HLA-DRA
HLA-DQA1
AGER
HLA-DPA1
CTLA-4
NOTCH4
PRTN3
HLA-DPB1
COL11A2
RXRB
HSD17B8
RING1

Figure S3. Gene ontology enrichment analysis of GPA (A), MPO-ANCA vasculitis (B), PR3-ANCA vasculitis (C), and ANCA-associated vasculitis (D). The gene set comprised the GWAS discovered SNP genes as well as noteworthy SNP genes identified in meta-analyses of observational studies and GWAS meta-analyses. Borderline SNPs ($5.0\times10^{-8} < p < 0.05$) from GWASs that were noteworthy were also included.

A

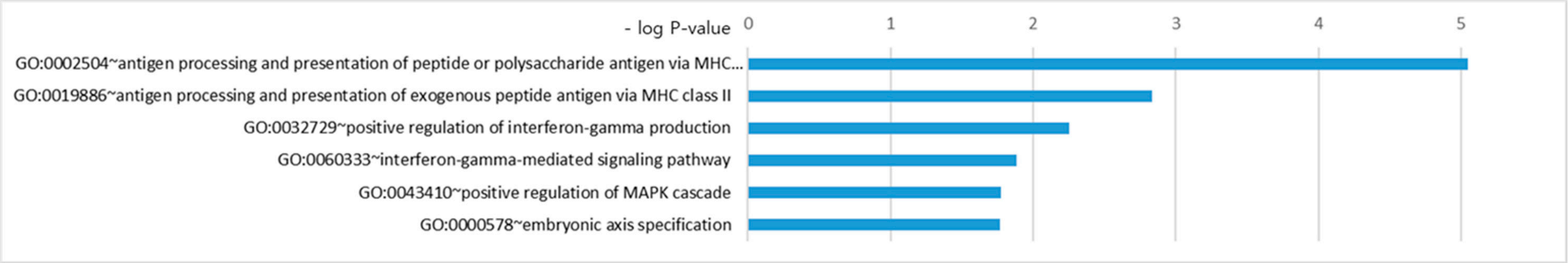

B

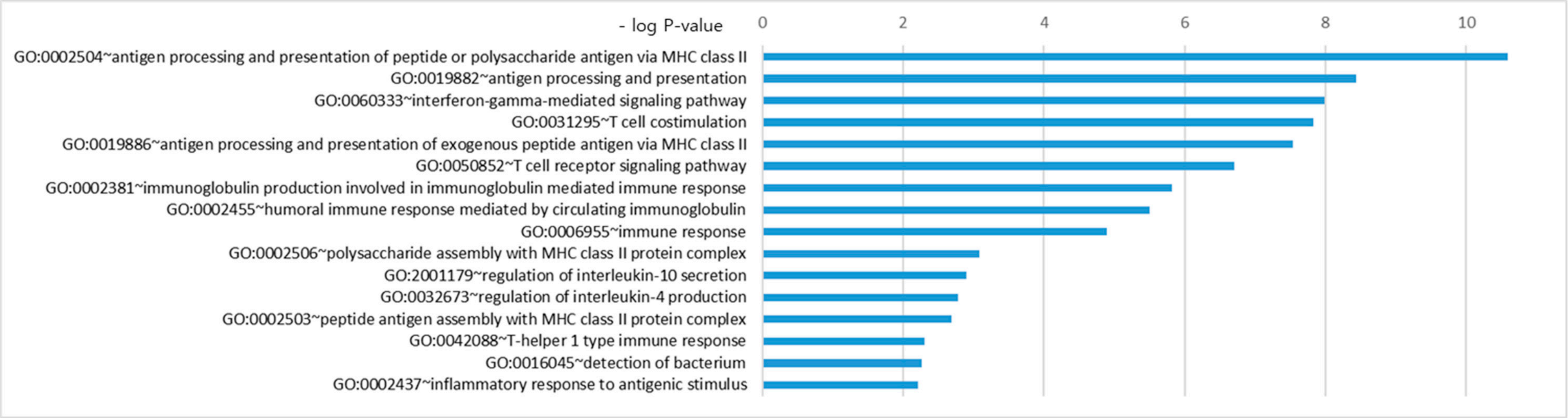

C

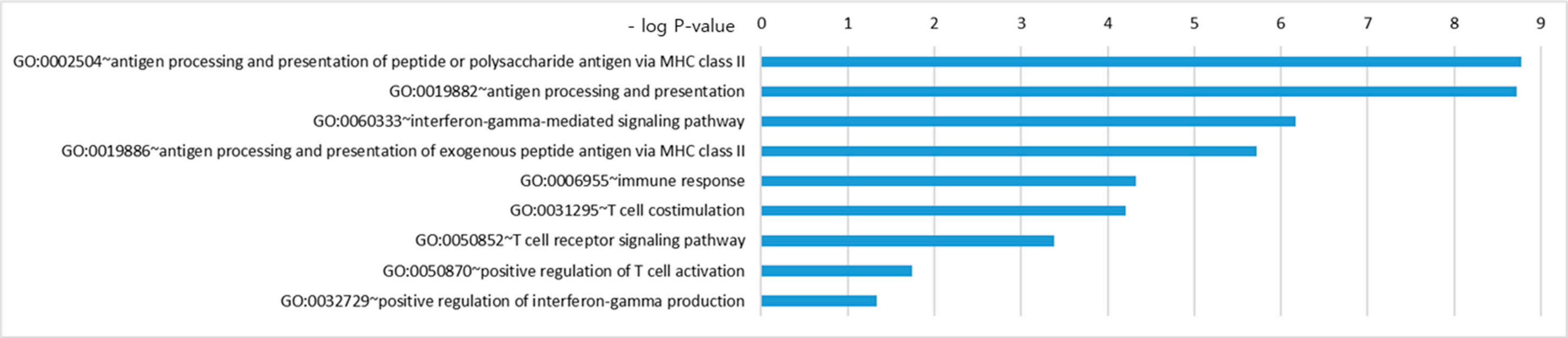

- log P-value
0
1
2
3
4
5
6
7
8
9
GO:0002504~antigen processing and presentation of peptide or polysaccharide antigen via MHC class II
GO:0019882~antigen processing and presentation
GO:0060333~interferon-gamma-mediated signaling pathway
GO:0019886~antigen processing and presentation of exogenous peptide antigen via MHC class II
GO:0006955~immune response
GO:0031295~T cell costimulation
GO:0050852~T cell receptor signaling pathway
GO:0050870~positive regulation of T cell activation
GO:0032729~positive regulation of interferon-gamma production

D

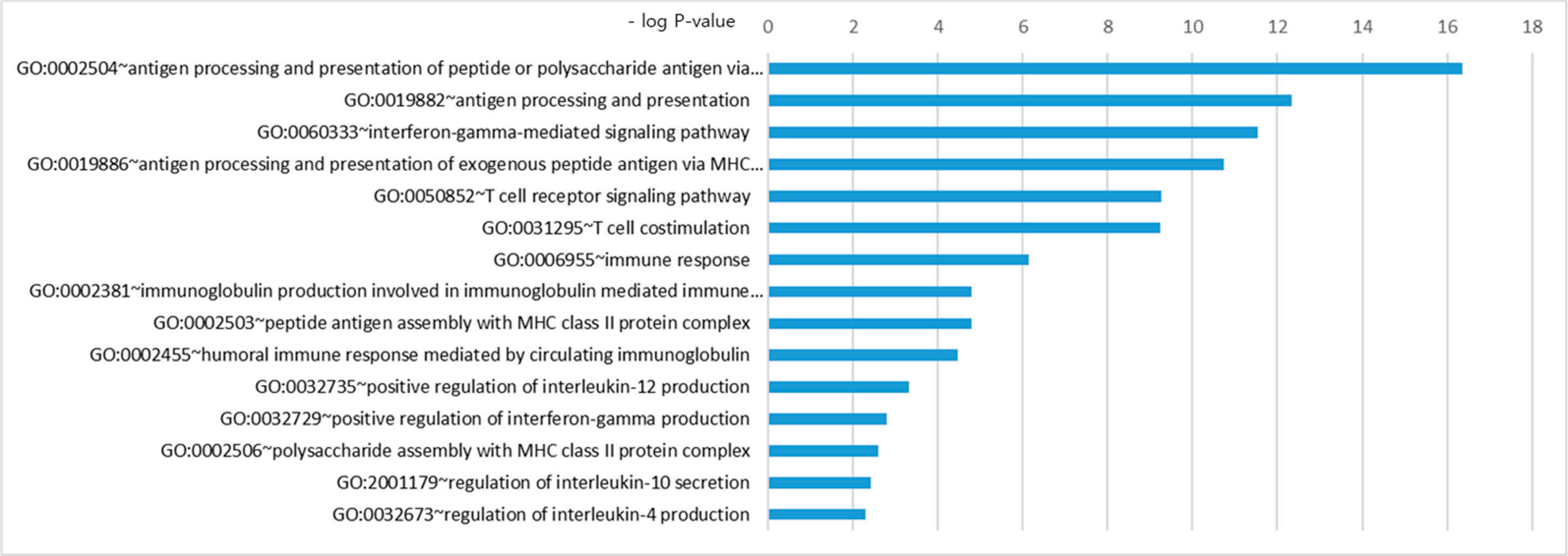

- log P-value
0
2
4
6
8
10
12
14
16
18
GO:0002504~antigen processing and presentation of peptide or polysaccharide antigen via...
GO:0019882~antigen processing and presentation
GO:0060333~interferon-gamma-mediated signaling pathway
GO:0019886~antigen processing and presentation of exogenous peptide antigen via MHC...
GO:0050852~T cell receptor signaling pathway
GO:0031295~T cell costimulation
GO:0006955~immune response
GO:0002381~immunoglobulin production involved in immunoglobulin mediated immune...
GO:0002503~peptide antigen assembly with MHC class II protein complex
GO:0002455~humoral immune response mediated by circulating immunoglobulin
GO:0032735~positive regulation of interleukin-12 production
GO:0032729~positive regulation of interferon-gamma production
GO:0002506~polysaccharide assembly with MHC class II protein complex
GO:2001179~regulation of interleukin-10 secretion
GO:0032673~regulation of interleukin-4 production

References

[1] Xie G, Roshandel D, Sherva R, Monach PA, Lu EY, Kung T, et al. Association of granulomatosis with polyangiitis (Wegener's) with HLA-DPB1*04 and SEMA6A gene variants: evidence from genome-wide analysis. Arthritis Rheum 2013; 65(9): 2457-68.

[2] Merkel PA, Xie G, Monach PA, Ji X, Ciavatta DJ, Byun J, et al. Identification of Functional and Expression Polymorphisms Associated With Risk for Antineutrophil Cytoplasmic Autoantibody-Associated Vasculitis. Arthritis Rheumatol 2017; 69(5): 1054-1066.

[3] Lee YH, Choi SJ, Ji JD, Song GG. CTLA-4 and TNF-α promoter-308 A/G polymorphisms and ANCA-associated vasculitis susceptibility: a meta-analysis. Mol Biol Rep 2012; 39(1): 319-26.

[4] Rahmattulla C, Mooyaart AL, van Hooven D, Schoones JW, Bruijn JA, Dekkers OM, et al. Genetic variants in ANCA-associated vasculitis: a meta-analysis. Ann Rheum Dis 2016; 75(9): 1687-92.

[5] Jung JH, Song GG, Lee YH. Meta-Analysis of Associations Between Interleukin-10 Polymorphisms and Susceptibility to Vasculitis. Immunol Invest 2015; 44(6): 553-65.

[6] Chung SA, Xie G, Roshandel D, Sherva R, Edberg JC, Kravitz M, et al. Meta-analysis of genetic polymorphisms in granulomatosis with polyangiitis (Wegener's) reveals shared susceptibility loci with rheumatoid arthritis. Arthritis Rheum 2012; 64(10): 3463-71.

[7] Lee YH, Choi SJ, Ji JD, Song GG. The protein tyrosine phosphatase nonreceptor 22 C1858T polymorphism and vasculitis: a meta-analysis. Mol Biol 2012; 39(8):8505-11.
